# Impact of the ‘FUNBALL’ Programme on Severe Injuries Among Young Male Football Players: A Secondary Analysis from a Cluster-Randomised Controlled Trial

**DOI:** 10.1186/s40798-025-00945-3

**Published:** 2025-11-27

**Authors:** Rilind Obërtinca, Rina Meha, Ilir Hoxha, Bujar Shabani, Tim Meyer, Karen aus der Fünten

**Affiliations:** 1 Department of Physiotherapy, University of Gjakova “Fehmi Agani”, Gjakova, Kosovo; 2 Institute of Sports and Preventive Medicine, Saarland University, 66123 Saarbrücken, Germany; 3 Dartmouth Institute for Health Policy and Clinical Practice, Lebanon, NH USA; 4 Evidence Synthesis Group, Prishtina, Kosovo; 5 Faculty of Medicine, University of Prishtina, Prishtina, Kosovo; 6 IFSTTAR, LBMc, Université Claude Bernard, Lyon, France; 7 Department of Orthopaedic Surgery, Johns Hopkins University School of Medicine, Baltimore, MD USA

**Keywords:** Adolescent football, Severe injuries, Injury prevention, Prevention programme, ‘FUNBALL’

## Abstract

**Background:**

‘FUNBALL’ is a new multi-component exercise-based injury prevention programme designed specifically for youth football players. Its efficacy in reducing the overall number of injuries has been previously reported. The aim of this study was to investigate the effects of the ‘FUNBALL’ programme in reducing the incidence of severe injuries (absence from training/match ≥ 28 days) in young male football players.

**Methods:**

55 football teams from Kosovo, 21 in the Under-15, 22 in the Under-17, and 12 in the Under-19 age groups, were cluster-randomly assigned to the intervention or the control group. The intervention group performed the ‘FUNBALL’ programme after their usual warm-up at least twice per week. The control group followed their usual training routine. Teams were followed for one football season (August 2021–May 2022). The outcome for the present analysis is severe injuries.

**Results:**

The overall incidence rate (IR) was 0.31/1000 football hours in the intervention group and 0.62/1000 football hours in the control group. Players in the age group of the Under-19s sustained the highest number of severe injuries (IR 0.77/1000 football hours). The overall number of severe injuries was significantly reduced by 49% (incidence rate ratio (IRR) 0.51; 95% CI 0.28–0.91; *P* = 0.02), with a 63% reduction in those that occurred during training (IRR 0.37; 95% CI 0.15–0.87; *P* = 0.02). When analyzed by age group, only the Under-17s showed a significant reduction of 76% (IRR 0.24; 95% CI 0.06–0.82; *P* = 0.02). The low number of subgroup injuries prevented statistical significance. However, a promising protective effect was observed. Severe knee injuries were reduced by 62%. By injury type, sprains or ligament injuries were reduced by 67%, and meniscus or cartilage lesions by 58%. Overuse/growth-related injuries were reduced substantially by 85%.

**Conclusion:**

The ‘FUNBALL’ programme showed a large efficacy in reducing the incidence of severe injuries in young male football players. Considering that these injuries cause the longest absence from football, it is recommended to implement the programme at least twice per week to exert a preventative effect.

**Trail Registration Number:**

Clinical trials NCT05137015.

**Supplementary Information:**

The online version contains supplementary material available at 10.1186/s40798-025-00945-3.

## Key Points

- The present study reports data on the epidemiology of severe injuries among young football players and the efficacy of the ‘FUNBALL’ programme in reducing the risk of severe injuries.
- The incidence of severe injuries increased with age; the most frequent affected location was the knee, the most common injury types were fractures and ligament sprains; and the longest time losses were associated with ACL ruptures.
- The ‘FUNBALL’ programme showed a large efficacy in reducing the incidence of severe injuries in young male football players.

**Supplementary Information:**

The online version contains supplementary material available at 10.1186/s40798-025-00945-3.

## Introduction

Similar to other age groups, adolescent football is injury-prone. Severe injuries account for 9.9% to 37% of all injuries [1–4]. A recent meta-analysis reported higher incidences of severe injuries in female youth football players in comparison to males: 1.25 injuries per 1000 football hours vs. 0.78 injuries per 1000 football hours, respectively [5]. These numbers are similar to the data reported for professional adult football players [6, 7], but higher compared to children [8]. Furthermore, Light et al. [1] reported the most common severe diagnoses observed in youth football, which included thigh muscle strain/tear/cramp (*n* = 38, 17%), ankle sprain/ligament injury (*n* = 37, 16%), knee bone injury (not fracture) (*n* = 21, 9%), and hip/groin muscle strain/tear/cramp (*n* = 14, 6%).

Football-related injuries negatively affect health and performance. Therefore, injury prevention has become a top priority in recent decades. The efficacy of various football-specific injury prevention programmes (IPPs) has been investigated in previous studies. However, despite considerable research conducted in different age groups, the evidence for reducing the risk of severe injuries in youth football is limited, but promising: the ‘FIFA 11+’ programme reported a risk reduction regarding severe injuries (rate ratio (RR) 0.55; 95% confidence interval (CI) 0.36–0.83) [9], the neuromuscular training programme revealed a reduction in ACL injuries in adolescent football players (RR 0.36; 95% CI 0.15–0.85) [10]. The ‘11 + kids’ proved to be effective in reducing severe injuries in children’s football (hazard ratio (HR) 0.26; 95% CI 0.10–0.64) [11, 12]. 

A new multi-component, exercise-based IPP, called ‘FUNBALL’, was investigated in young male football players (13 to 19 years old). Through two cluster-randomised controlled trials (cluster-RCTs), its efficacy in preventing football-related injuries [13] and improving various cognitive abilities [14] was revealed. The players in the intervention group improved in working memory, visual learning, visual motor control, attention, psychomotor function, memory, and executive function [14]. Enhancements in cognitive abilities such as attention, executive function, and visuomotor control may help prevent injuries in youth football players by enhancing hazard detection, decision-making, and motor coordination [15, 16]. 

In the current study, we aimed to perform a secondary analysis of the cluster-RCT [13] regarding the efficacy of the ‘FUNBALL’ programme in reducing the risk of severe injuries, resulting in an absence of ≥ 28 days from football, which are particularly concerning as they can lead to long-term participation restriction, dropout from youth football, or reduced progression opportunities.

## Methods

### Study Design and Participants

The design of the study was a two-armed cluster-RCT. The study was conducted in the youth football leagues in Kosovo. The study protocol was registered in the ClinicalTrials.gov registry (identifier: NCT05137015) and was approved by the ethics committee of the Kosovo Chamber of Physiotherapists (identifier 2020/368). A detailed methodological description is given in the publication of the main cluster-RCT [13]. Briefly, 21 football clubs (with 70 teams) from Kosovo were invited to participate in our study, with their Under-15, Under-17, and Under-19 male teams. All teams participated in youth football leagues organised by the Football Federation of Kosovo. The inclusion criteria for our trial were that teams had to (1) be officially registered in the national football association and (2) train at least twice per week in addition to a football match. Teams that were already using a structured IPP were excluded. All the teams that enrolled in the study (55 teams with 1253 players) were randomised into an either intervention (INT) or control (CON) group. The INT group performed the ‘FUNBALL’ programme at least twice per week. The CON group followed their usual training routine. Teams were followed for one football season (nine months).

### Intervention

The ‘FUNBALL’ program was specifically designed for youth football players. It involved collaboration between the research community and end-users at every stage of development [17, 18]. The final version included exercises aimed at improving muscle strength and neuromuscular control. Specifically, the following exercise categories were included: (1) balance; (2) core stability; (3) hamstring muscle eccentrics; (4) gluteal muscle activation; (5) plyometrics; (6) running/sprinting; and an optional category of (7) games. All exercises were organised into five or six progressive levels of increasing difficulty. The programme was performed after their usual warm-up and took about 15–20 min to complete after familiarisation. Research staff visited each intervention team three to four times per season to monitor programme’s execution and quality. The complete and the short version of the intervention programme are available as supplementary material.

### Outcome Measures

For the present study, the following outcomes were investigated: the overall number of football-related severe injuries (causing an absence from training/match of ≥ 28 days) that occurred during the season; region-specific severe injuries of the lower limbs (hip/groin, thigh, knee, lower leg, ankle, and foot); injury type (fracture, sprain/ligament injury, muscle injury, lesion of the meniscus or cartilage, tendon injury, other bone injuries, dislocation/subluxation, and other); and injury mechanism (contact, non-contact, and overuse injuries).

### Data Collection Procedures and Definitions

We collected data during the football season from August 2021 to May 2022 using the data collection procedures and definitions according to the consensus statement on injury definitions and data collection procedures [19]. Throughout the competitive season, the coaches or team’s physiotherapists reported to the research assistants the following data: the team exposure hours, the programme execution, and any football-related injuries that occurred. Thereafter, the assistants contacted the injured players or their parents if they were under age to obtain information regarding the injury and filled out a standardized injury registration form adopted by Rössler et al. [11]. The vast majority of severe injuries were registered based on a detailed medical examination. All injuries were screened for plausibility by two medical doctors (BS and KadF, co-authors of this study), who independently verified each diagnosis reported by clubs, players, or parents for accuracy and consistency.

### Statistical Analysis

A pre-trial sample size calculation was performed for the cluster-RCT [13] using G*Power (Version 3.1) with a two-sided z-test to generate the required sample size. Thereafter, we aimed to recruit 806 football players, 403 per arm. Descriptive statistics were reported for baseline characteristics. Continuous variables (age, height, weight, BMI, and football experience) were reported as mean and standard deviation (SD). We utilized the intention to treat principle to calculate incidence rate ratios (IRR) along with 95% confidence intervals (CIs) for each outcome and compared them between the intervention and control groups. A Poisson regression model was used to account for the cluster effect, with the team being considered as the cluster variable. Training, match, and total football exposure hours were calculated according to previous research [11, 12, 19, 20]. The injury incidence (IR) with 95% CI was calculated according to the formula IR=(n/e)×1000; ***n***, number of football injuries, and ***e***, total exposure time) [9]. Injury burden was calculated as the number of days lost to injury per 1000 h of football (“injury incidence x mean absence per injury”) [21]. Mean days lost per injury were reported with standard deviation (SD). Two-tailed p values were considered significant when the α-error had a level of less than 0.05. The statistical analyses were conducted using Stata statistical software Version 17 BE (Stata Corp., Texas, United States), and MS Excel.

## Results

### Participants, Exposure and Injury Characteristics

82% of the randomised teams completed the study (*n* = 45/55). Players in the two groups (mean age 15.3 years) were similar in terms of baseline characteristics (Table 1). Football exposure hours are presented in Table 1. A total of 50 severe injuries was recorded during the season. They accounted for 16% of all injuries (*n* = 319). Players in the age group of the Under-19s sustained the highest number of severe injuries (*n* = 21, 42%, incidence rate (IR) 0.77/1000 football hours), followed by the Under-17s (*n* = 17, 34%, IR 0.41/1000 football hours), and the Under-15s (*n* = 12, 24%, IR 0.31/1000 football hours). In both groups (INT and CON), the knee was the most frequently affected region (*n* = 18, 36%, IR 0.18/1000 football hours). Fractures were the most common injury type in the INT group (*n* = 5, 29.4%, IR 0.09/1000 football hours), while sprain/ligament injuries were most common in the CON group (*n* = 9, 27.3%, IR 0.17/1000 football hours). Anterior cruciate ligament (ACL) ruptures caused the longest time loss resulting in 1321 (mean, 264.2) days across five incidents (one in the INT group and four in the CON group) (Table 2). Further data on characteristics of severe injuries are presented in Tables 1 and 2.

**Table 1:** Player and injury characteristics of the intervention and control groups

	Intervention group (mean ± SD)	Control group (mean ± SD)
*Player characteristics*		
Teams Under-15 Under-17 Under-19	23 9 9 5	22 8 9 5
Number of players Under-15 Under-17 Under-19	524 206 206 112	503 204 190 109
Age (years)	15.22 (1.6)	15.28 (1.6)
Height (cm)	171 (9.1)	172 (7.9)
Weight (kg)	60.2 (8.6)	60.5 (8.3)
BMI (kg/m^2^)	20.4 (1.5)	20.3 (1.7)
Football experience^a^ (years)	5.0 (1.8)	4.9 (1.6)
*Exposure characteristics*		
Total exposure hours	53,454	52,938
Match exposure hours	9017	8666
Training exposure hours	44,437	44,273
*Injury characteristics*		
Number of severe injuries	17	33
Number of match injuries	10	14
Number of training injuries	7	19
Injury burden (days)	17	46

cm, centimetre; kg, kilogram; BMI, body mass index; SD, standard deviation

^a^Football experience taking into account the years since the players has trained at least three times per week

**Table 2:** Diagnosis, frequency and total days lost to injury of severe injuries (≥ 28 days)

Diagnosis	Number of injuries	Time loss (days)	Mean days lost (SD)
ACL rupture	5	1321	264.2 (17.9)
Meniscus tear	5	271	54.2 (16.9)
Osgood Schlatter syndrome	3	174	58 (5.2)
Toe fracture	4	163	40.8 (6.1)
Clavicle fracture	2	150	75 (12.7)
Osteitis pubis	3	132	44 (7.5)
Hip joint synovitis	2	127	63.5 (31.8)
Forearm fracture	3	99	33 (4.6)
Hamstring strain	3	96	32 (3)
Lower leg fracture	1	96	96 (n/a.)
Ankle sprain	3	91	30.3 (2.1)
Colateral lat/med ligament sprain	2	74	37 (5.7)
Groin strain	2	68	34 (7.1)
Knee pain	2	64	32 (1.4)
Ankle dislocation	1	63	63 (n/a.)
Shoulder dislocation	1	61	61 (n/a.)
Elbow dislocation	1	47	47 (n/a.)
Achilles tendon tendinitis	1	46	46 (n/a.)
Knee joint synovitis	1	43	43 (n/a.)
Elbow (olecranon) fracture	1	41	41 (n/a.)
Metatarsal I fracture	1	41	41 (n/a.)
Calf muscle (gastrocnemius) strain	1	36	36 (n/a.)
Lower leg pain (growth related)	1	35	35 (n/a.)
Quadriceps strain(rectus femoris)	1	32	32 (n/a.)
Total	50	3371	67.4 (68.3)

### Effects of the Intervention Programme on Severe Injuries

There was a significantly lower incidence of severe injuries in the INT group, with a 49% reduction (incidence rate ratio (IRR) 0.51; 95% CI 0.28–0.91; *P* = 0.02). Severe injuries occurred in training were reduced by 63% (IRR 0.37; 95% CI 0.15–0.87; *P* = 0.02). The analysis according to age groups showed a significant reduction in the incidence of severe injuries only among the Under-17 players (IRR 0.24; 95% CI 0.06–0.82; *P* = 0.02).

Due to a low number of events, the incidence of subgroups of severe injuries did not reach any significant group difference (Table 3). Regarding the body locations, severe knee injuries were reduced by 62%. The most commonly prevented injury types were sprains or ligament injuries, reduced by 67%, and meniscus or cartilage lesions, reduced by 58%. Overuse/growth-related were reduced by 85%. The injury burden was 17 days lost per 1000 h in the INT group and 46 days lost per 1000 h in the CON group.

**Table 3:** Injury characteristics and injury mechanisms of the severe injuries (time loss ≥ 28 days)

	Intervention group (*n* = 17 severe injuries)		Control group (*n* = 33 severe injuries)		IRR (95% CI)	*P* value
	No. of injuries	IR	No. of injuries	IR		
*Total severe injuries*	17	0.31	33	0.62	0.51 (0.28–0.91)	0.02*
Match severe injuries	10	1.10	14	1.61	0.69 (0.30–1.54)	0.36
Training severe injuries	7	0.15	19	0.42	0.37 (0.15–0.87)	0.02*
Under-19’s severe injuries	10	0.73	11	0.81	0.90 (0.38–2.11)	0.80
Under-17’s severe injuries	3	0.15	14	0.64	0.24 (0.06–0.82)	0.02*
Under-15’s severe injuries	4	0.19	8	0.45	0.44 (0.13–1.45)	0.17
*Location*						
Knee	5	0.09	13	0.24	0.38 (0.13–1.06)	0.06
- *ACL*	1	0.01	4	0.07	0.25 (0.02–2.21)	0.21
Foot/toe	1	0.01	4	0.07	0.25 (0.02–2.21)	0.21
Hip/groin	1	0.01	3	0.05	0.33 (0.03–3.17)	0.33
Thigh	2	0.03	2	0.03	0.99 (0.14–7.03)	0.99
Lower leg/Achilles tendon	1	0.01	3	0.05	0.33 (0.03–3.17)	0.33
Ankle	1	0.01	3	0.05	0.33 (0.03–3.17)	0.33
Shoulder/clavicle	1	0.01	2	0.03	0.50 (0.04–5.46)	0.56
Lower back/sacrum	1	0.01	2	0.03	0.50 (0.04–5.46)	0.56
Forearm	3	0.05	–	–	–	–
Elbow	1	0.01	1	0.01	0.99 (0.06–15.83)	0.99
*Type*						
Fracture	5	0.09	7	0.13	0.71 (0.22–2.22)	0.55
Sprain/ligament injury	3	0.05	9	0.17	0.33 (0.08–1.21)	0.09
Lesion of meniscus or cartilage	3	0.05	7	0.13	0.42 (0.11–1.64)	0.21
Muscle injury	3	0.05	5	0.09	0.59 (0.14–2.48)	0.47
Tendon injury	1	0.01	3	0.05	0.33 (0.03–3.17)	0.33
Dislocation/subluxation	2	0.03	1	0.01	1.98 (0.01–21.84)	0.57
Other	-	–	1	0.01	–	–
*Injury mechanism*						
Contact	7	0.13	13	0.24	0.53 (0.21–1.33)	0.18
Foul against injured player	4	0.07	7	0.13	0.57 (0.16–1.93)	0.36
Fouling another player	-	-	2	0.03	–	–
Duel	-	-	3	0.05	–	–
Other	3	0.05	1	0.01	2.97 (0.30–28.56)	0.34
Non-contact	7	0.13	12	0.22	0.58 (0.22–1.46)	0.24
Change of direction	1	0.01	3	0.05	0.33 (0.03–3.17)	0.33
Running	3	0.05	3	0.05	0.99 (0.20–4.90)	0.99
Jumping	3	0.05	5	0.09	0.59 (0.14–2.48)	0.47
Shooting	–	–	1	0.01	–	–
Ball	–	–	–	–	–	–
Overuse/growth-related	3	0.05	8	0.15	0.37 (0.09–1.14)	0.14

IR, incidence rates, are reported per 1000 h of football play and are unadjusted; IRR, incidence rate ratios, are adjusted for team; CI, confidence interval; *p < 0.05

## Discussion

### Severe Injuries in Young Football Players

The incidence of severe injuries reported by previous research conducted in youth football varied largely, ranging from 0.16 to 1.74/1000 football hours [1, 3, 4, 9, 22, 23]. In the present study, severe injuries accounted for 16% of all injuries, with an overall IR of 0.46 per 1000 football hours. This incidence was lower in comparison to studies that included the same population, youth male footballers. Overall IRs there ranged between 0.97 and 1.12 per 1000 football hours [1, 4]. This could be attributed to the ‘FUNBALL’ implementation, which lowered the incidence of severe injuries in the intervention group. However, when exclusively examining the control group [24], the incidence of severe injuries (IR of 0.62) was still lower than reported in the previously mentioned studies. One plausible explanation could be the level of play, as our study involved a lower playing level compared to the aforementioned research, which included participants from English Premier League academies. A higher playing level may increase competitiveness in training and matches, potentially contributing to a higher incidence of severe injuries. On the other hand, in our study, the incidence of severe injuries was comparable to that reported by Le Gall et al.^3^ (IR of 0.46 vs. IR of 0.47) in elite youth French footballers.

In terms of the location of severe injuries, the knee emerged as the most commonly affected region, accounting for 36% (IR of 0.09 and 0.24 per 1000 football hours in the INT and CON, respectively) of all cases in both groups. Potentially, this high frequency of severe knee injuries in comparison to other locations might have been influenced by the playing surface. All participating teams trained and played their matches on artificial turf. The risk arising from artificial turf specifically for knee ligament injuries has recently been well documented [25, 26]. There were higher rates for anterior cruciate ligament (ACL) and posterior cruciate ligament (PCL) injuries during competitions on artificial turf versus natural grass [25, 26]. 

### Efficacy of the ‘FUNBALL’ on Overall and Region-Specific Severe Injuries

The ‘FUNBALL’ reduced the incidence of overall severe injuries by 49% [13, 27]. Given the long absence resulting from severe injuries, the teams of the intervention group experienced a higher player availability. This substantial reduction is clinically relevant due to its impact on player health, training consistency, and long-term athletic development [28]. 

Statistical significance was not reached for the secondary outcome variables, i.e. region-specific injury subcategories. This can be explained by the low number of injuries within the subgroups. Nonetheless, there seems to be a protective effect regarding various injuries. Regarding the locations, the most noticeable protective tendency was observed for severe knee injuries. A similar trend was shown for ACL ruptures in particular. These findings are clinically valuable, as ACL injuries have long-term implications, including joint instability and an increased risk of degenerative changes that can affect athletic participation and overall quality of life [29]. ‘FUNBALL’ contained categories that may have positively influenced the risk for knee/ACL injuries e.g., via gluteal and plyometric exercises. Rinaldi et al. [30] documented that the gluteal muscles play a critical role in maintaining a proper knee alignment, i.e., preventing a dynamic knee valgus during physical activities like walking, jogging, jumping, and landing. Additionally, Al Attar et al. [31] demonstrated that including plyometric activities in IPPs significantly lowered the incidence of ACL injuries.

A protective trend for other lower limb region-specific (hip/groin, lower leg, ankle, and foot/toe) severe injuries was observed. Previous research emphasized the association between impaired core stability and the occurrence of lower limb injuries in an athletic population [32]. Apart from the other categories within ‘FUNBALL’, it might be possible that core stability exercises have also played a role in influencing this result.

### Efficacy of the ‘FUNBALL’ on Types of Severe Injuries

Regarding the injury type, sprain/ligament injuries had a noticeably lower incidence in the intervention group compared to the control group. Fractures were the most common in the intervention group while sprain/ligament injuries in the control group. Fractures are usually contact-related and therefore more difficult to prevent. Moreover, protective tendencies were shown for other severe injury types, i.e. muscle, tendon, and meniscus or cartilage injuries. Previous research reported the efficacy of IPPs on the aforementioned injury types if certain exercises, such as balance, core stability, and hamstring eccentrics exercises are included. ‘FUNBALL” contains all those categories. Specifically, the effectiveness of balance exercises in reducing ankle ligament injuries in football players was previously demonstrated [33–35]. Moreover, numerous studies observed the efficacy of hamstring eccentrics on preventing hamstring injuries [36–38]. However, a recent meta-analysis with a more detailed methodological evaluation that included calculating prediction intervals concluded that the efficacy of Nordic hamstring exercises remains uncertain and inconclusive [39]. For the first time in connection with IPPs, the ‘FUNBALL’ included sprinting exercises. The latest evidence reported that they may mitigate hamstring injury risk [40]. 

### Efficacy of the ‘FUNBALL’ on the Mechanism of Severe Injuries

The ‘FUNBALL’ programme aimed to reduce non-contact injuries such as sprain/ligament injuries that may present a greater potential for prevention. As mentioned before, ‘FUNBALL’ was successful in reducing contact-related injuries by 47%. Even though the vast majority of IPPs target non-contact injuries, some reduction of the risk of contact injuries through IPPs had already been reported, too [41]. Similar to previous multi-component exercise-based IPPs, ‘FUNBALL’ included strength exercises that focused on core stability and lower extremity strength. Moreover, exercises that included activities such as hopping, jumping, and landing were part of the programme. They potentially enhance balance, lower leg strength and functional leg stability, thereby improving the body’s ability to absorb and counteract external forces, such as those induced from physical contacts.

A tendency towards enhanced protection in the intervention group was also identified for overuse/growth-related injuries. Growth-related issues were previously reported as major issues in children’s and youth football, especially Osgood-Schlatter Disease [8, 42]. In our study, we found that 6% (*n* = 3; INT = 1 and CON = 2 cases, respectively) of all severe injuries were attributed to this specific diagnosis. From a clinical perspective, such cases often require long-term modification of football-related activities, structured rehabilitation, and careful load management to reduce recurrence and prevent long-term functional limitations.

### Strengths and Limitations

The present study reports a detailed secondary investigation of data from a large cluster-randomised trial. Specifically, for the present study, the main strengths are the design and methodology, which followed the best available research practice. A thorough clarification of the protocols for injury classification and injury definitions was carried out for the research assistants before the season started. The accuracy of the diagnosis and the injury surrounding circumstances of severe injuries is high as 92% of the injured players were seen by a medical doctor. Finally, two medical doctors unaware of the clubs’ assignment to the intervention or control group checked the plausibility of the injury information.

On the other hand, this study also has some limitations. The decision to return after the injury was a very important one. The influence of the parents on the decision to return could have influenced the real time loss. In some instances, the doctor approved the return to team training, but some parents chose to keep their children on the sidelines for an extended period. However, this may increase data variance but hardly constitutes a bias. The strength of the clinical recommendations from the study is negatively affected by the lack of female footballers in our trial. Additionally, the lack of individual exposure hours presents another limitation in our study. This made it impossible to analyse the individual’s compliance with the intervention. This type of analysis was only possible on a team basis. Furthermore, the sample size was calculated for overall injury statistics but not for severe injuries, and particularly not for their subcategories. However, conducting a study solely focused on severe injuries or their subcategories would require an enormous number of participants. Finally, additional factors could affect the generalizability of this secondary analysis in youth football players. These include regional differences, variations in climate and atmospheric conditions, and differences in club governing body rules, all of which can influence injury risk.

### Summary and Future Research

Over the course of a full football season, the ‘FUNBALL’ programme reduced the incidence of severe injuries by 49% among youth male players. Given the significant burden of such injuries, this finding highlights the importance of systematic prevention strategies. To achieve meaningful benefits, the programme should be performed at least twice per week. It is recommended that ‘FUNBALL’ is used as part of the regular training routine, as coach-led implementations is likely to ensure consistent adherence. Movement quality would also benefit in this supervised condition. Both aspects positively impact on the long-term effectiveness. Our findings indicate a higher efficacy in younger age categories, the Under-15s and Under-17s. This suggests that age-specific adaptations may be necessary to optimize outcomes across different developmental stages. While secondary outcomes also showed a protective trend, further research involving larger cohorts and longer follow-up periods is warranted, particularly to assess the programme’s impact on less frequent but severe injuries, such as ACL ruptures. Finally, given the promising results in male adolescents, future studies should explore the implementation and effectiveness of the ‘FUNBALL’ programme in youth female football players.

## Supplementary Information

Below is the link to the electronic supplementary material.

Supplementary Material 1.

Supplementary Material 2

Supplementary Material 3.

## Data Availability

The datasets used and/or analysed during the current study are available from the corresponding author on reasonable request.
