# Supplementary material for: Impact of the ‘FUNBALL’ Programme on Severe Injuries Among Young Male Football Players: A Secondary Analysis from a Cluster-Randomised Controlled Trial
Source: Sports Med Open. 2025 Nov 27;11:151. doi: 10.1186/s40798-025-00945-3 (PMC12660612; doi:10.1186/s40798-025-00945-3)
Supplement: Supplementary file 1 — Supplementary Material 1. [file 40798_2025_945_MOESM1_ESM.pdf]

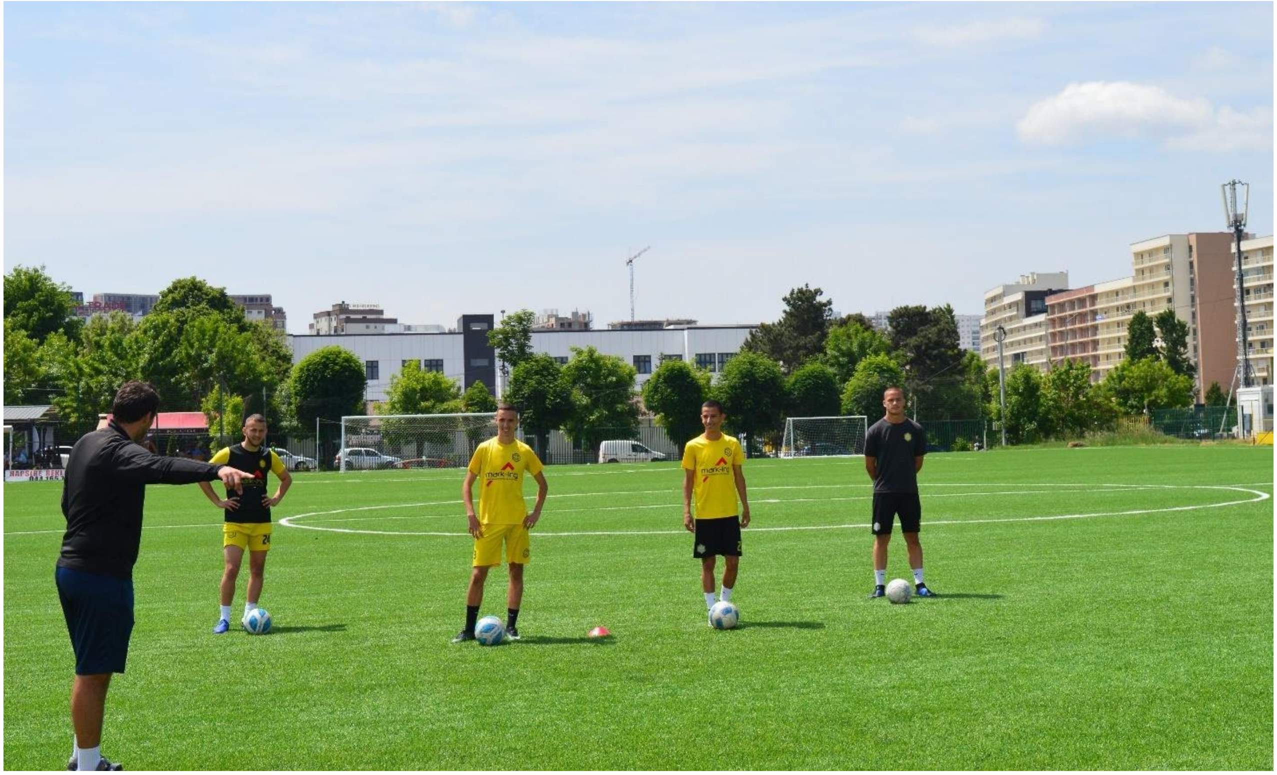

Injury Prevention Programme

‘FUNBALL’

Manual for coaches

Development and conceptual design

M. Sc. Rilind Obërtinca, PhD. Cand. (sports physiotherapy)

Institute of Sports and Preventive Medicine, Saarland University, Saarbrücken, Germany

Karen aus der Fünten, MD. (orthopaedic surgeon, sports medicine, manual therapy), MChiro

Institute of Sports and Preventive Medicine, Saarland University, Saarbrücken, Germany

Prof. Dr. Tim Meyer, MD, PhD. (sports medicine)

Institute of Sports and Preventive Medicine, Saarland University, Saarbrücken, Germany

In cooperation with

M. Sc. Rina Meha, PhD. Cand. (cognitive neuropsychology)

Institute of Sports and Preventive Medicine, Saarland University, Saarbrücken, Germany

Prof. Dr. Sabine Schaefer (movement science and sport psychology)

Institute of Sports Science, Saarland University, Saarbrücken, Germany

M. Sc. Elon Berisha (football coach)

Football Federation of Kosovo

TABLE OF CONTENTS

1. Introduction 4
2. General outline, content and structure of the program 4
3. General guidelines of the program 5
4. Equipment 5
5. Posture 5

6. **PROGRAM EXERCISES** 6
    6.1. **Balance** 6
        a. One leg stance 6
        b. Y-Balance 10
    6.2. **Core stability** 14
        a. Plank and side plank 14
        b. Straight arm plank 18
    6.3. **Hamstring muscles eccentrics** 22
        a. Nordic Hamstrings 22
        b. Hamstring walk outs 26
    6.4. **Gluteal muscle activation** 30
        a. Head, Shoulder, Hip, Knee, Ankle 30
        b. Squat Lunges 35
    6.5. **Plyometric** 39
        a. Forward jumps 39
        b. Skater jumps 42
    6.6. **Running/sprinting** 45
        a. Diagonal running/sprinting 45
        b. Forward running/ssprinting 49
    6.7. **Games** 53
        a. Tic-Tac-Toe 53
        b. Header Game 54
        c. Dribbling Game 55

1. Introduction

Football is the most popular sport in the world, with 260 million male and female active participants, including ~113,000 FIFA registered professional players. Playing football is fun and can provide many health benefits, however it also presents a high injury risk. Therefore, this program has been created by international professionals and aims to reduce football injuries for football players aged 13+.

2. General outline, content and structure of the program

The execution of the program takes around 15 minutes and should be used at least twice per week. The program will be performed in the training sessions, after the usual warm-up. The warm-up should prepare the players for intense exercises, such as jumping and sprinting.

The program is based on scientific evidence that has previously shown good efficacy on injury prevention in football. The exercise categories address 7 aspects:

1. Balance
2. Core stability
3. Hamstring muscle eccentrics
4. Gluteal activation
5. Plyometric
6. Running/Sprinting
7. Games

The games are included with the aim to increase the attractiveness of the program.

Each category contains 2 exercises and the coach is free to decide which one to choose in every training session. All exercises are organized in five or six levels with increasing difficulty (physically and cognitively). If players can perform a level with the correct technique, it is the coach’s decision to move to the next level. The instructions for the players should be short, clear, and concise.

3. General guidelines of the program

- The levels should be completed in the designated order. No level should be skipped. A player can train at the 3rd level in one exercise while he is at the 1st level in another exercise.
- A player can start with the next level if the exercise has been carried out correctly in three successive training sessions.
- The breaks between the sets should last approximately 20-30 sec (when applicable).
- If the cone colours are replaced for numbers, the replacement becomes random and changes in every training session.
- If numbers replace the cone colour, the numbers used should be from 1-99.
- If calculations are used in the exercises the resulting number should be singular between 1 and 9.
- 3-digit numbers are used in a few levels, when players are asked to react only to the last number.

4. Equipment

For the execution of this program, the team needs basic training equipment, such as balls, cones, hurdles, and training kits.

5. Posture

A highly important aspect of the program is good posture retention. Proper posture keeps the body structures in optimal alignment, which increases the effect of the exercises while reducing the risk of suffering an injury. The coaches should continuously pay attention to the correct posture of the footballers while performing the exercises. The correct posture and possible errors for each exercise are described in detail alongside the exercise description. Correct posture is more important than speed when performing each exercise.

PROGRAM EXERCISES

6.1. Balance exercises

a. Single leg stance

Correct exercise posture:

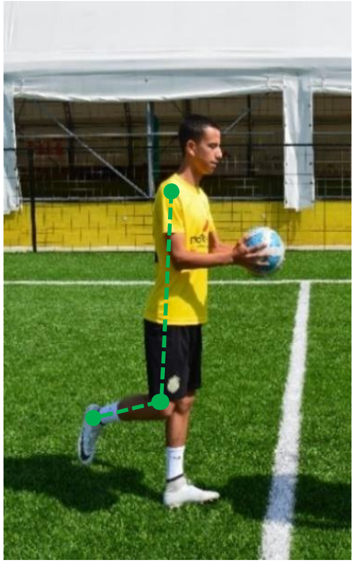

- ✓ Lift one leg off the ground at 90°.
- ✓ The position of the head, neck, back, and thighs should be in one line.
- ✓ The head is straight and the gaze is forward.
- ✓ Distribute the weight on the standing leg evenly between the heel and the balls of the foot.

Make sure to correct these errors:

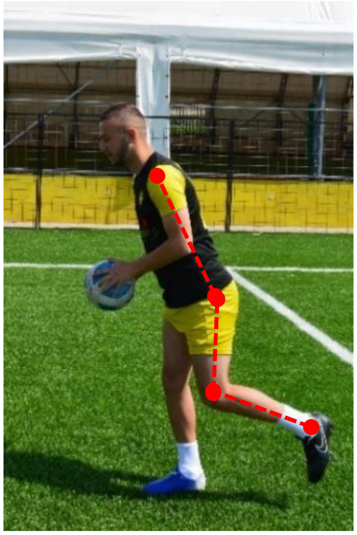

- × Do **not** bend the hips.
- × Do **not** let the knee move inwards.
- × Do **not** lift the forefoot or heel off the ground.
- × Do **not** lean forward.

Starting position: players are in pairs. They stand on one leg facing each other about 2 arm lengths apart. The players look straight ahead. They bend the knee of the swing leg 90°. Both players carry a ball in their hands.

Equipment needed: 1-2 balls per pair.

Level 1

Action: move the ball around the belly.

Repetition: 2x 30 seconds (on each leg).

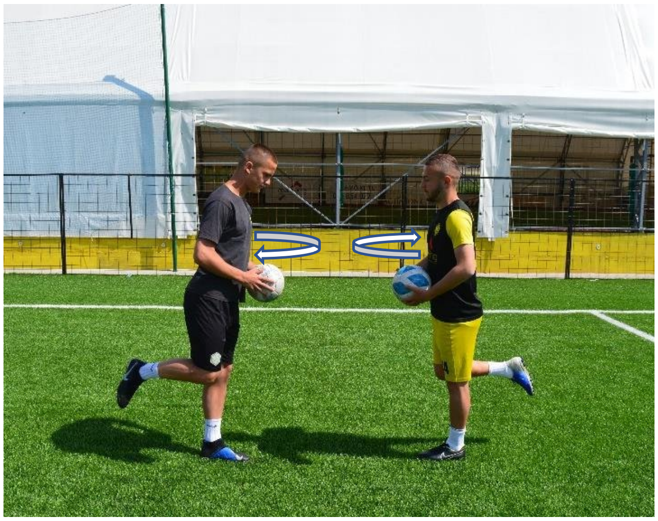

Level 2

Action: same as level 1. The eyes are closed.

Repetition: 2x 30 seconds (on each leg).

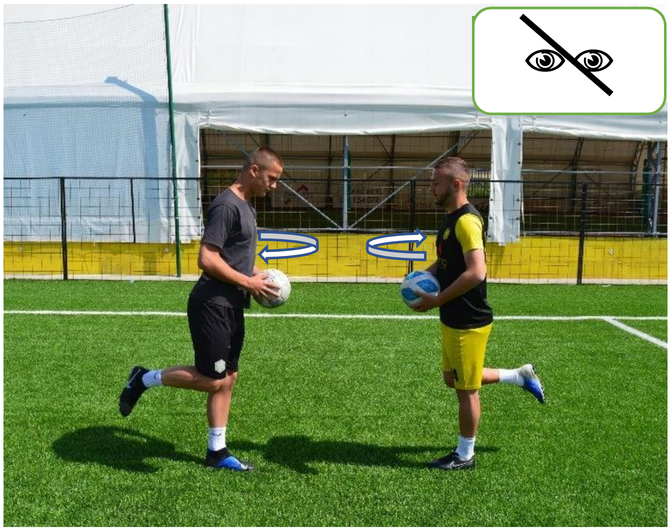

Level 3

Action: using 1 ball, hand it to the partner at different body levels. Coach randomly instructs e.g., head, shoulder, hip, knee and feet.

Repetition: 2x 30 seconds (on each leg).

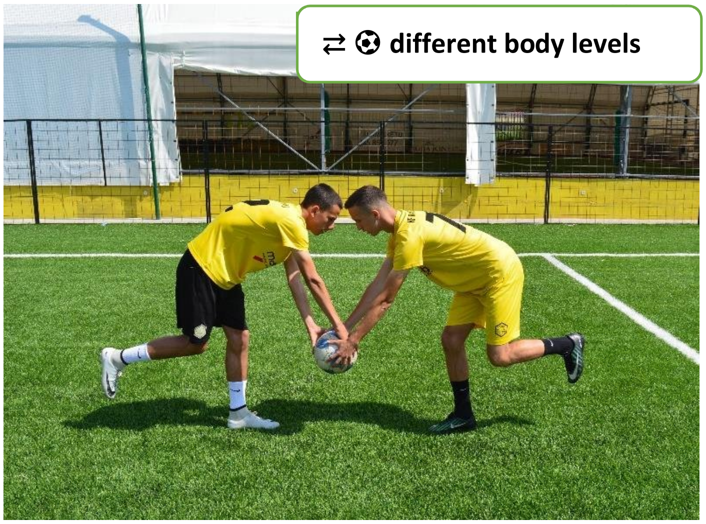

Level 4

Action: hand the ball to the partner in the opposite direction from the coach's instruction. When instruction is "UP" hand the ball "DOWN", and vice versa. Same applies for "LEFT" and "RIGHT".

Repetition: 2x 30 seconds (on each leg).

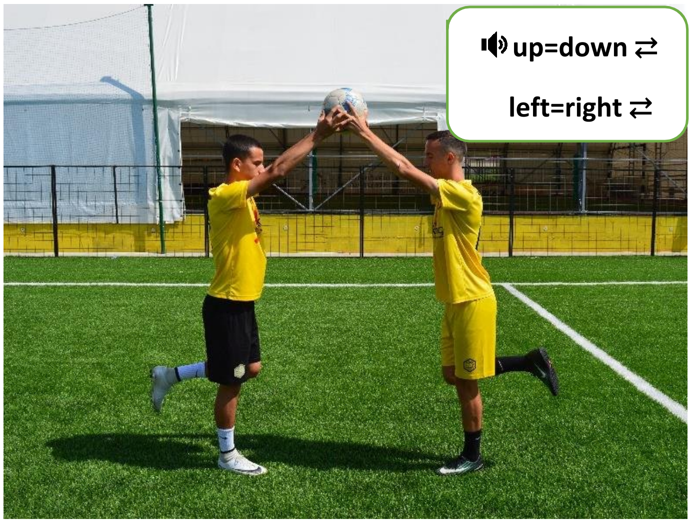

Level 5

Action: the eyes are closed. Hand the ball straight to the partner at upper body level.

Explanation: *the players pass the ball when the coach instructs "PASS". Players should be of similar height.*

Repetition: 2x 30 seconds (on each leg).

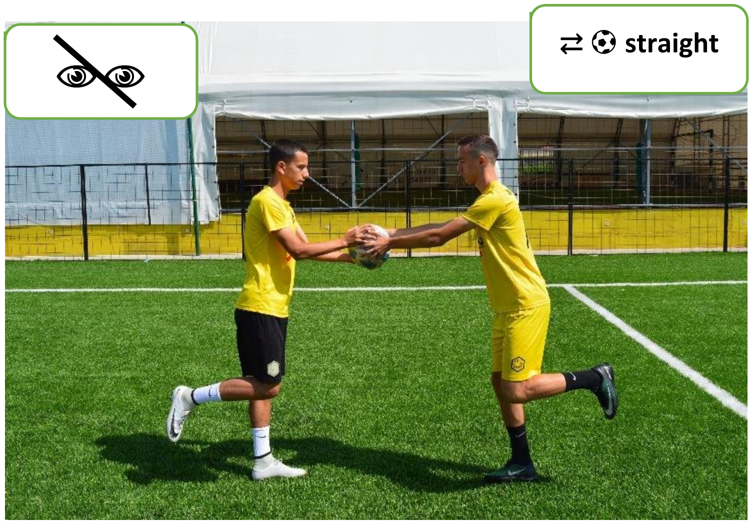

Level 6

Starting position: the distance between players is increased to 2 meters, (distance can be increased over time to 3m and 4m). There is one ball per pair.

Action: using their feet, players pass the ball to each other. The other player catches the ball with his hands.

Repetition: 2x 30 seconds (on each leg).

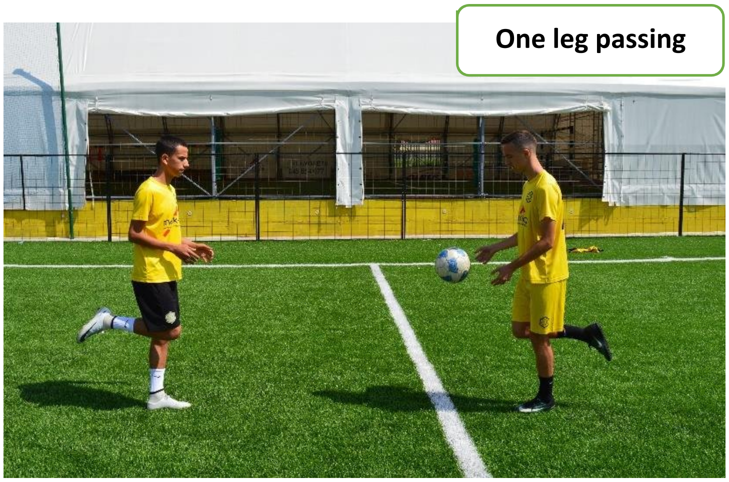

b. Y-Balance

Correct exercise posture:

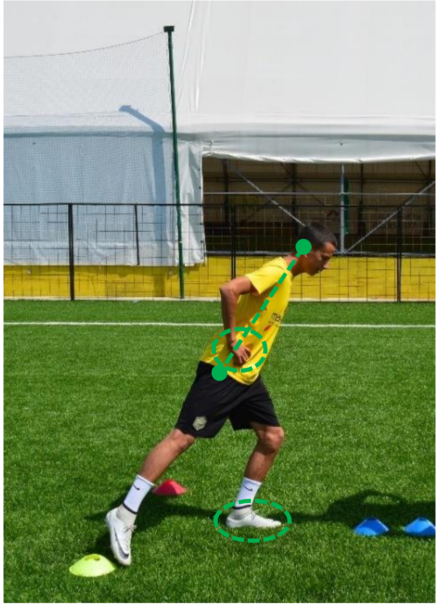

- ✓ Lift one leg off the ground.
- ✓ Other foot is always in contact with the ground.
- ✓ The position of the head, neck, back should be in one line.
- ✓ Hands rest on the hips.
- ✓ Distribute the weight on the standing leg evenly between the heel and the balls of the foot.

Make sure to correct these errors:

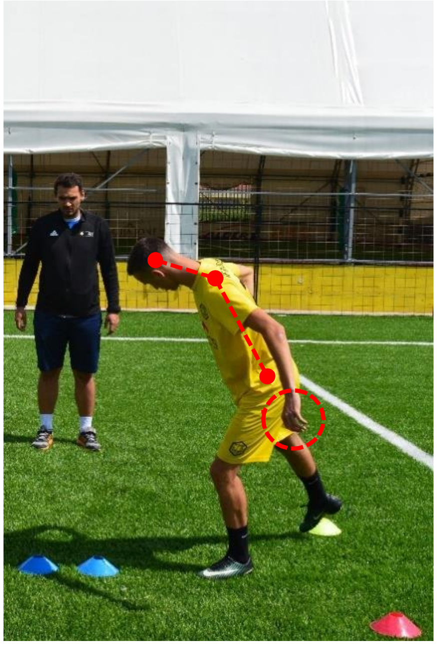

- × Do **not** let the knee move inwards (into valgus).
- × Do **not** lift the forefoot or heel.

Starting position: players are in pairs. They stand on one leg facing each other. The players look straight ahead. Each player stands in the middle of 3 cones or any objects available e.g., clothing of different colours. The cones are placed in a 'triangle shape' on the ground 80cm apart. Cone distance increases over levels. One cone is in front and 2 cones are behind the respective player. The colour of the cones of the players are mirroring one another, *(e.g., if the "red" colour is behind and to the left for player 1, it is also behind and to the left for player 2)*. Players remain on the same leg until the end of the set.

Equipment needed: 6 cones of 3 different colours per pair.

Level 1

Action: coach instructs the colour. The players reach the respective cone with their foot of the swing leg.

Repetition: 3x 6 repetitions (on each leg).

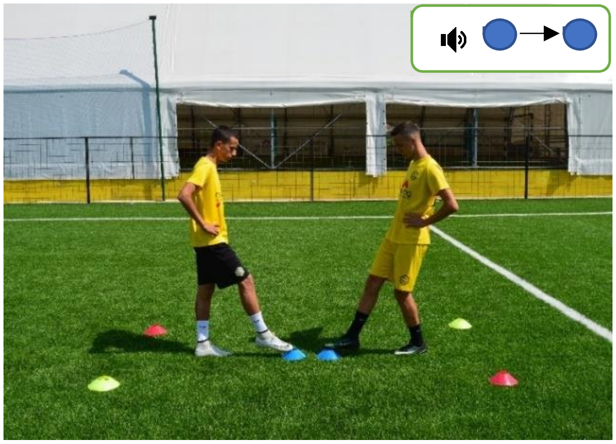

Level 2

Action: same as level 1. The eyes are closed.

Repetition: 3x 6 repetitions (on each leg).

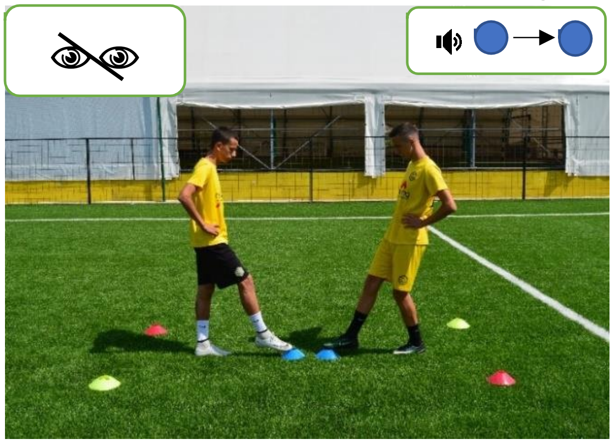

Level 3

Cone distance: increased to 90cm.

Action: player 1 reaches one colour with their foot of the swing leg. Player 2 reflects the move of player 1.

Repetition: 3x 6 repetitions (on each leg).

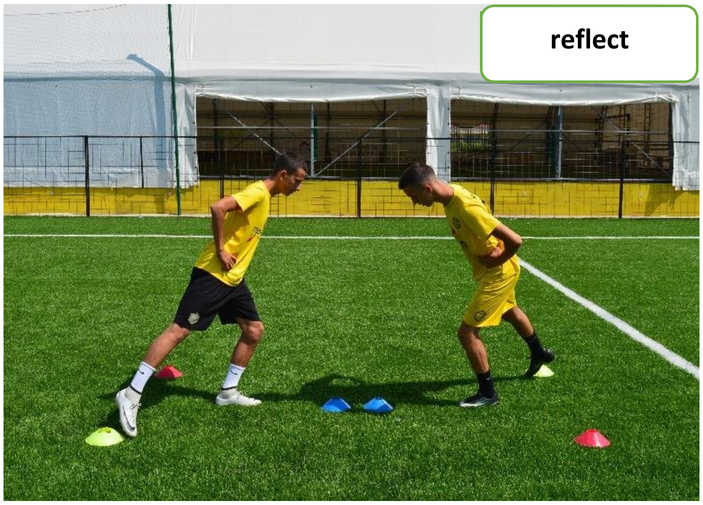

Level 4

Action: coach replaces the colour of the cones for numbers, e.g. *"red"=1, "blue"=2, "yellow"=3*, and instructs the numbers. The players reach the respective cone with their foot of the swing leg.

Repetition: 3x 8 repetitions (on each leg).

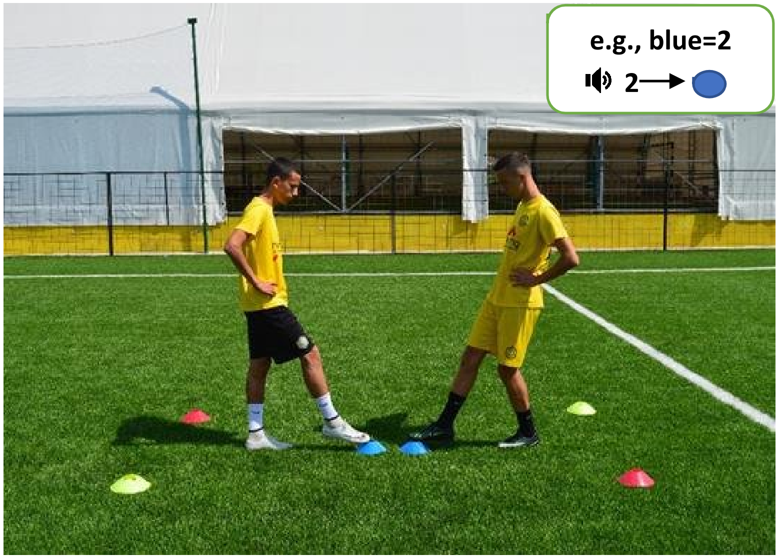

Level 5

Cone distance: increased to 1m.

Action: same as level 4. The eyes are closed.

Repetition: 3x 8 repetitions (on each leg).

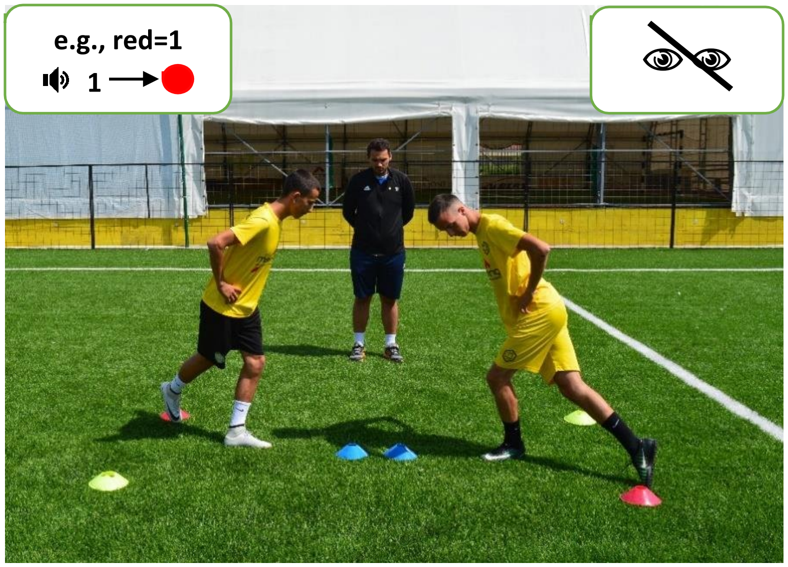

Level 6

Action: coach uses calculations to have the respective instructed numbers, e.g., *(1= "red", 2= "blue", 3= "yellow"): 3-2=" red", 9-7=" blue", 1+2=" yellow").* The players reach the respective cone with their foot of the swing leg.

Repetition: 3x 8 repetitions (on each leg).

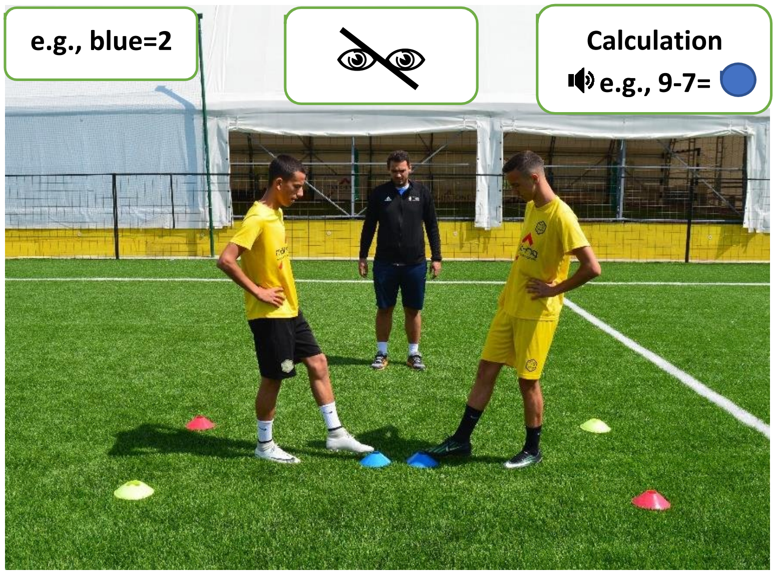

6.2. Core Stability exercises

a. Plank and Side Plank

Correct exercise posture:

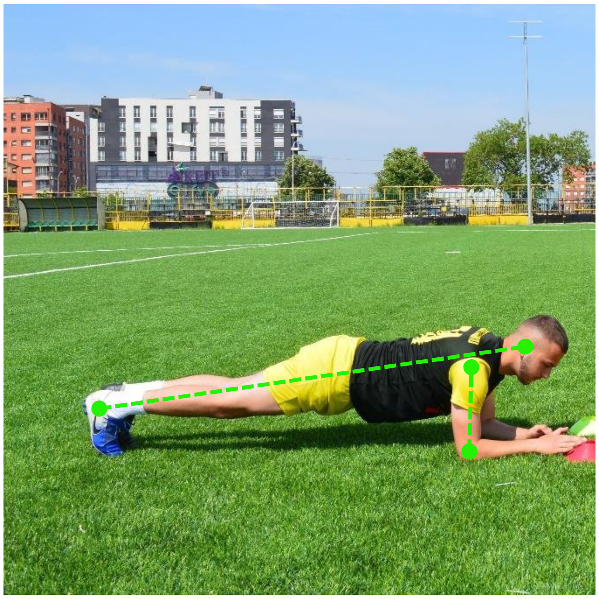

- ✓ Place forearms on the ground.
- ✓ Forearms are parallel to your body about shoulder width apart.
- ✓ Elbows are aligned below shoulders.
- ✓ The position of the lower legs, thighs, back, neck and head should be in one line.
- ✓ Stay on the toes.
- ✓ The gaze is fixed on the ground.

Make sure to correct these errors:

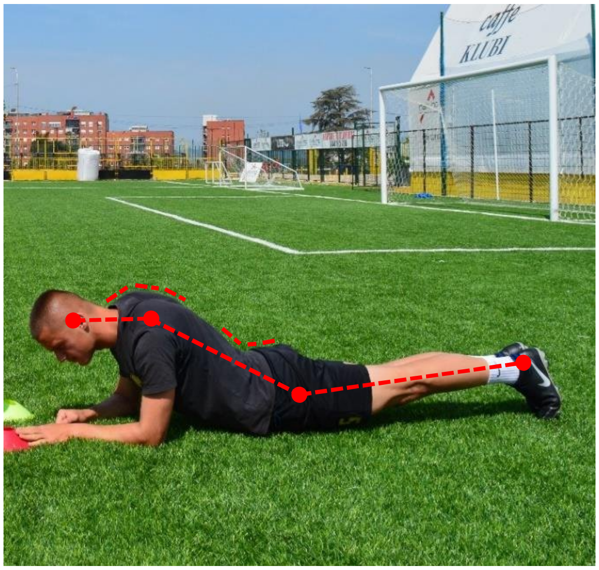

- × Do **not** drop the head.
- × Do **not** drop the lower back.
- × Do **not** raise the buttocks.
- × Do **not** let the pelvis shift sideways or up/down.

Starting position: the players are in pairs. They face each other in the plank and both side plank positions (on the left and right forearm) respectively. Two cones of different colours are placed in the middle of them, shoulder width apart. Distance of the players from the cones is one hand length.

Equipment needed: 3 cones of different colour per pair.

Level 1

Action: hold the plank positions. Players begin with a side plank, then turn to the front plank and finally to the other side plank position (=one set).

Repetition: 2x 20 seconds (on each plank position).

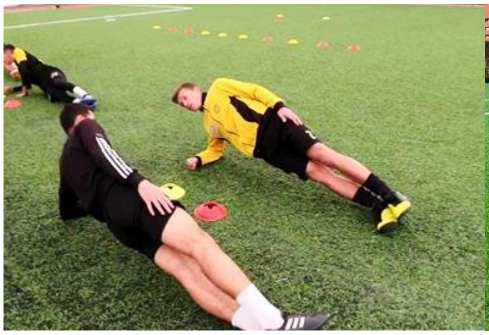

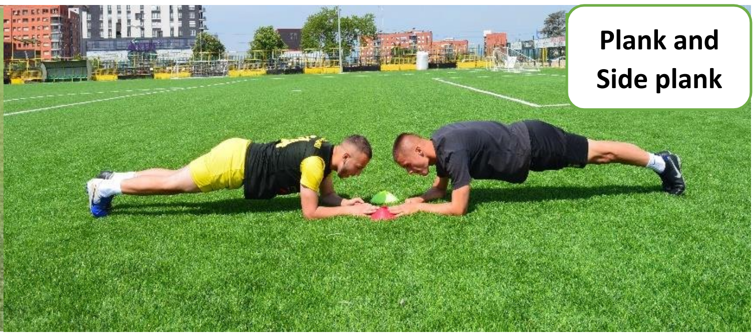

Level 2

Action: hold the plank positions. Coach instructs a colour. The players compete to touch that cone first. They lift the arm from the ground to touch the cone.

Repetition: 2x 20 seconds (on each plank position).

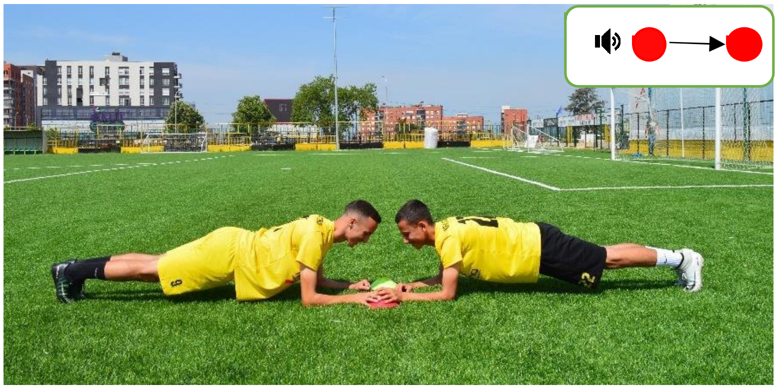

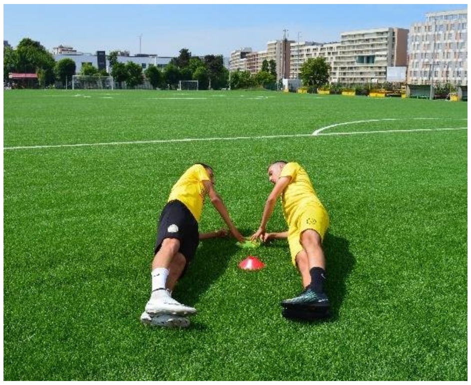

Level 3

Action: same as level 2. The players compete for the colour that was not instructed.

Repetition: 2x 30 seconds (on each plank position).

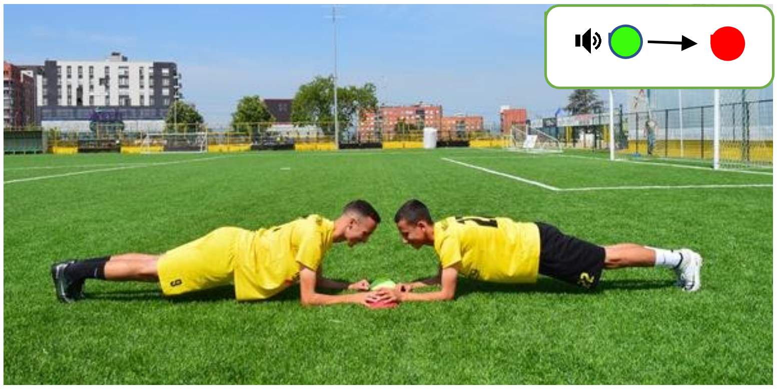

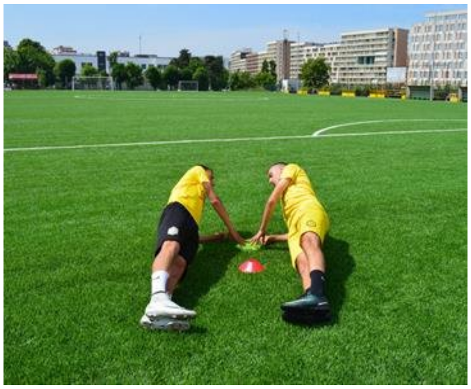

Level 4

Starting position: distance of the players from the cone is increased to an arm length in front plank position. In the side plank position distance between the players remains the same (1 hand length).

Action: same as level 3. The eyes are closed.

Repetition: 2x 30 seconds (on each plank position).

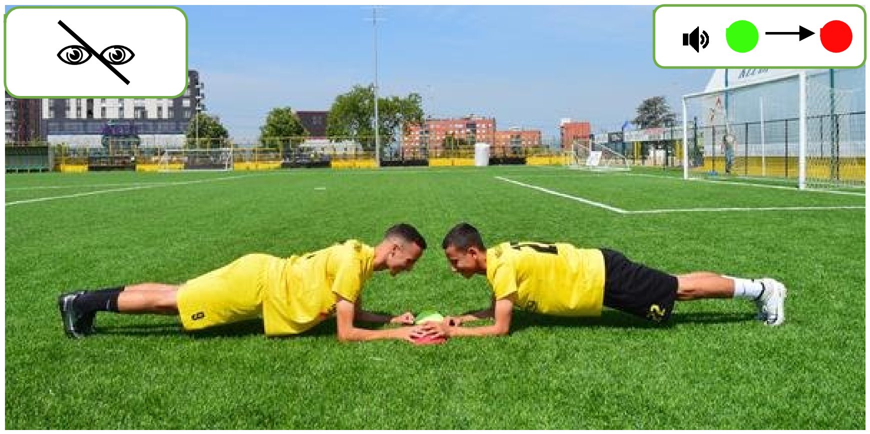

Level 5

Action: the eyes are closed. Hold the plank positions. Coach replaces the colour of the cones for numbers, *e.g., "red" =1, "yellow" =2.* Coach instructs numbers. The players compete for the colour that was not instructed.

Repetition: 2x 40 seconds (on each plank position).

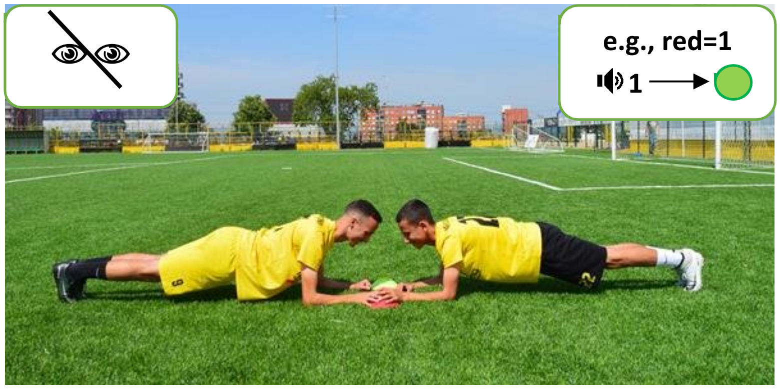

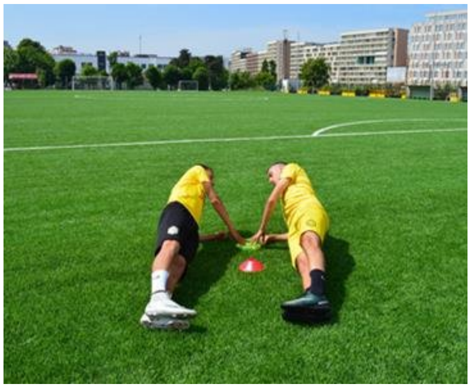

Level 6

Starting position: in front of players are now placed 3 cones of a different colour. The middle cone is placed in front of the players face, other cones one hand width apart on either side.

Action: hold the plank positions. Coach replaces the colour of the cones for numbers, e.g. *"red"=2, "blue"=4, "green"=6*. Coach instructs a three-digit number. Based on the last number of the instruction, players compete for the cone represented by that number.

Repetition: 2x 40 seconds (on each plank position).

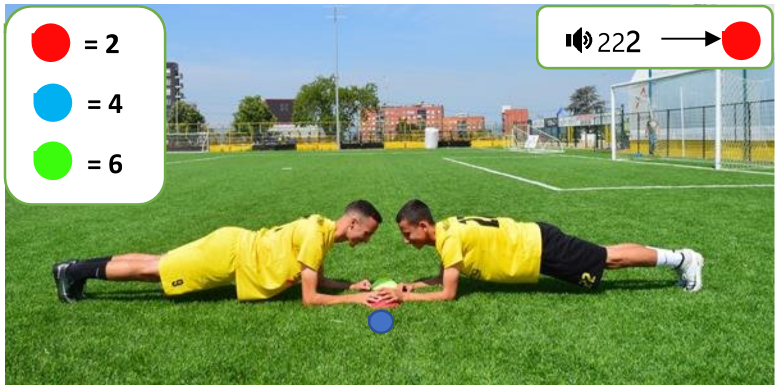

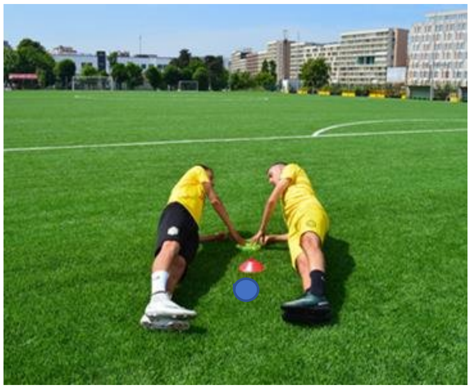

b. Straight Arm Plank

Correct exercise posture:

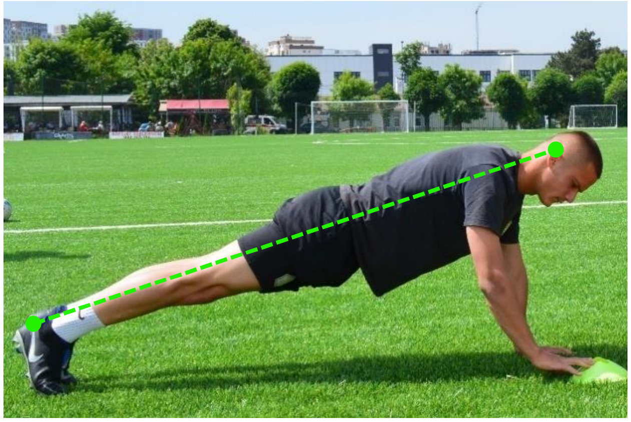

- ✓ Place hands on the ground.
- ✓ Wrists, elbows and shoulders should be in one line.
- ✓ Arms are perpendicular to the body about shoulder width apart.
- ✓ The position of the lower legs, thighs, back, neck and head should be in one line.
- ✓ Stay on the toes.
- ✓ The gaze is fixed on the ground.

Make sure to correct these errors:

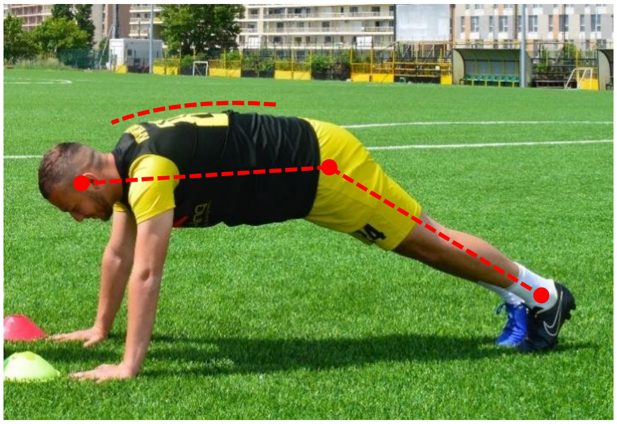

- × Do **not** drop the head.
- × Do **not** drop the lower back.
- × Do **not** raise the buttocks.
- × Do **not** let the pelvis shift sideways or up/down.

Starting position: the players are in pairs. They face each other in a straight arm plank position. Two (later three) cones of different colours are placed in between them. Distance of the players from the cones is one hand width.

Equipment needed: 3 cones of different colours per pair.

Level 1

Action: coach instructs the colour. Players compete to catch the cone first. At the end of the set the player with the lower score does three push-ups. If they are equal, both do three push-ups (continues in all levels).

Repetition: 2x 8 repetitions.

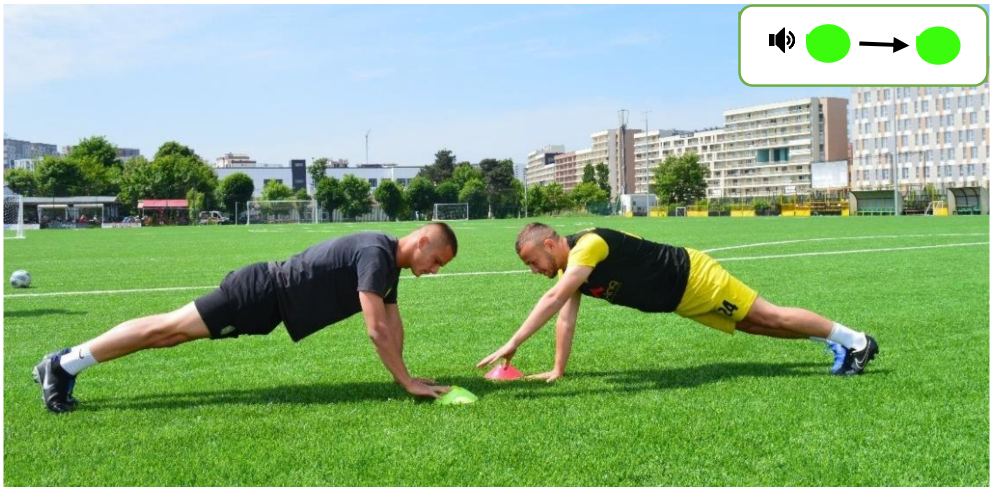

Level 2

Action: same as level 1. Players compete for the colour that was not instructed.

Repetition: 2x 8 repetitions.

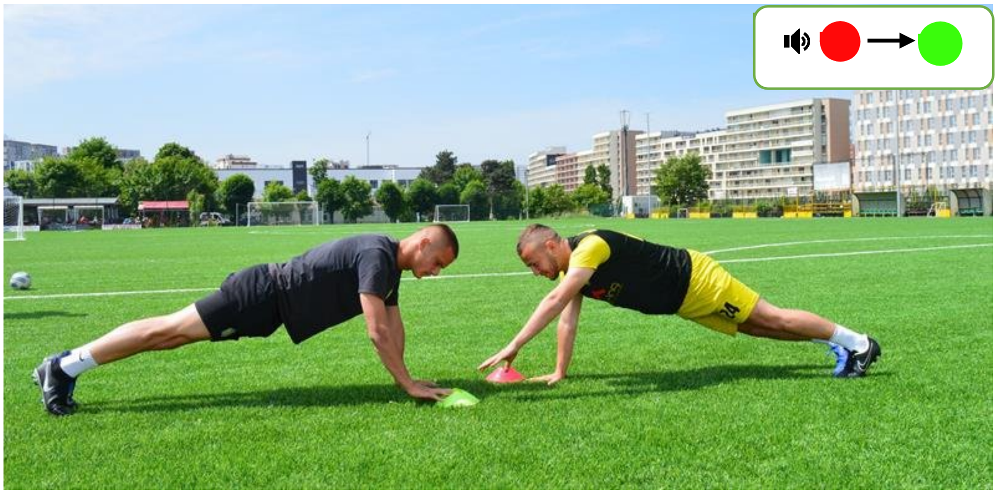

Level 3

Action: coach replaces the colour of the cones for odd and even numbers, *e.g., "red" =even, "green" =odd.* Coach instructs three-digit numbers. If the last number is odd, players compete for the cone representing odd numbers.

Repetition: 2x 10 repetitions.

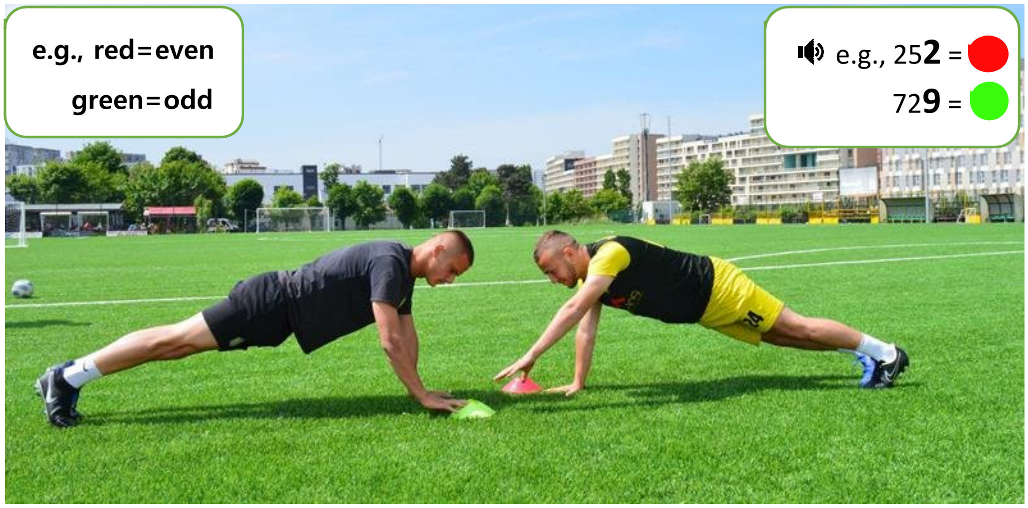

Level 4

Starting position: distance of the players from the cone is increased to an arm length from the cones.

Action: coach uses a calculation to have odd and even numbers. Players compete for the cone representing odd or even numbers.

Repetition: 2x 10 repetitions.

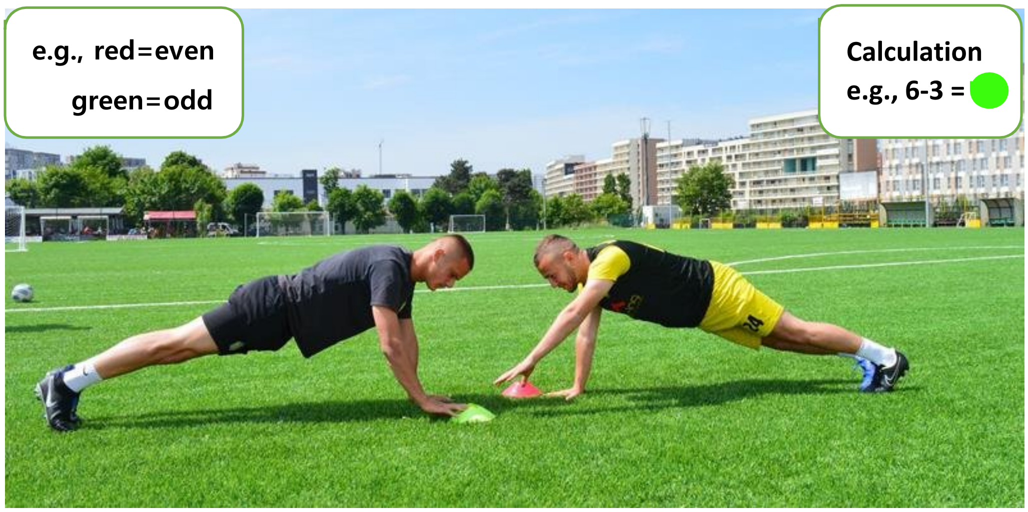

Level 5

Starting position: same as level 1. Three cones of different colours are placed in between them. The middle cone is placed in front of players, other cones one hand width apart on either side.

Action: coach instructs the colour. Players compete for the colour instructed.

Repetition: 2x 12 repetitions.

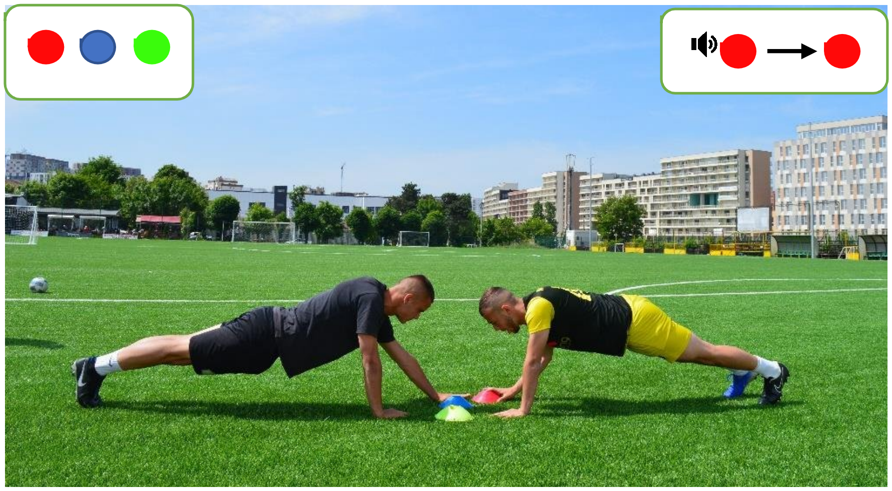

Level 6

Action: each colour of the cones represents a specific number told previously to the players, e.g., *"red" =3, "green" =5, and "blue" =9.* Coach instructs a three-digit number. Based on the last number of the instruction players compete for the cone represented by that number.

Repetition: 2x 12 repetitions.

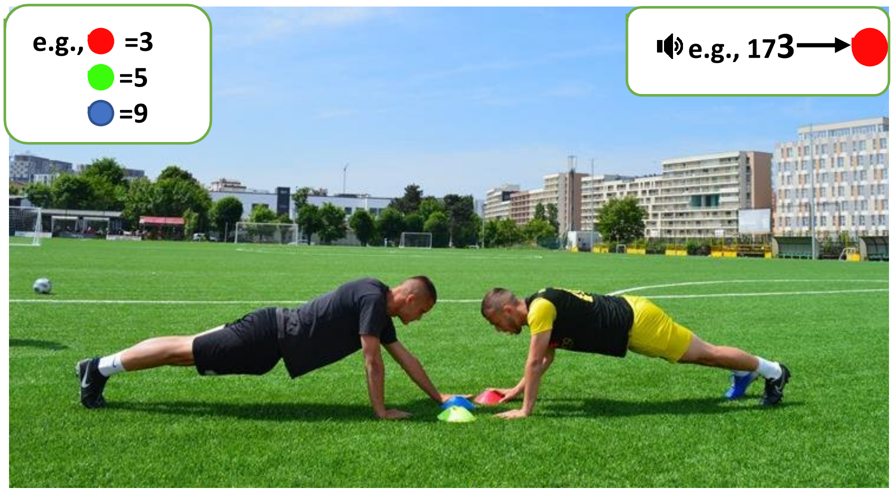

6.3. Hamstring muscles eccentrics

a. Nordic Hamstring

Correct exercise posture:

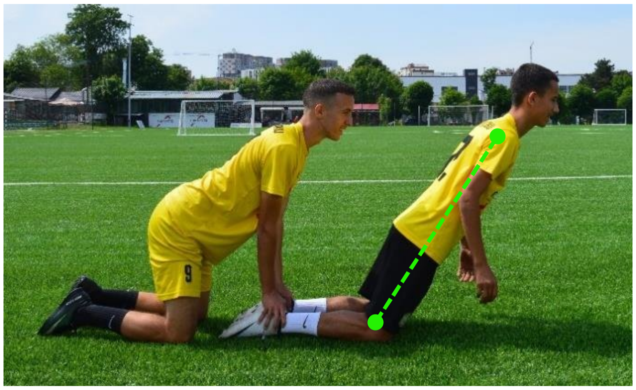

- ✓ Feet, lower legs, and knees stay in touch with the ground.
- ✓ Knees are about shoulder width apart.
- ✓ The position of the thighs, hips, back, neck and head should be in one line.

Make sure to correct these errors:

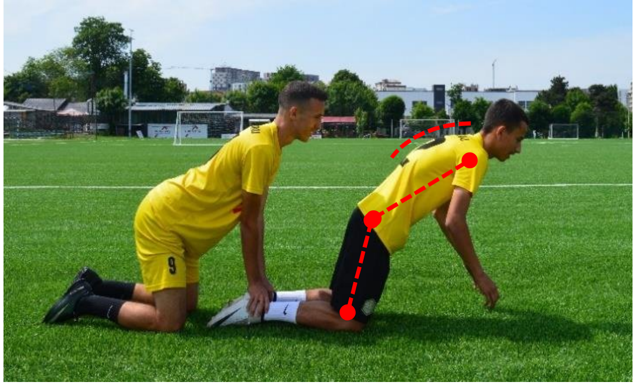

- × Do **not** drop the head.
- × Do **not** bend the hips.
- × Do **not** round the back.

Starting position: one player starts in a kneeling position with the upper body upright. The partner applies pressure to the athlete's heels/lower legs to ensure that the knees, lower legs and feet stay in touch with the ground throughout the movement.

Equipment needed: none.

Explanation: *players will change the level every 6 weeks.*

Level 1

Action: player leans forward in a straight line from head to knee and in a controlled manner and tries to avoid falling forward. If players cannot control the movement any further, they catch themselves on their hands.

Repetition: 1x 3-5 repetitions.

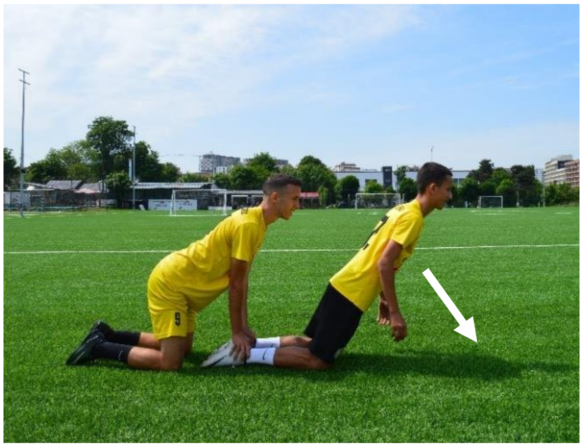

Level 2

Action: same as level 1.

Repetition: 1x 6-8 repetitions.

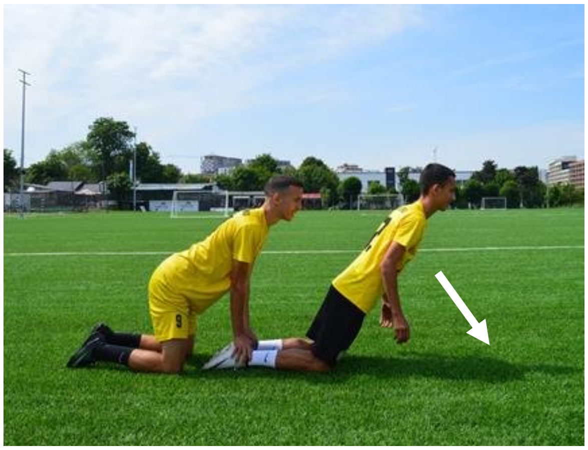

Level 3

Action: same as level 1.

Repetition: 1x 10-12 repetitions.

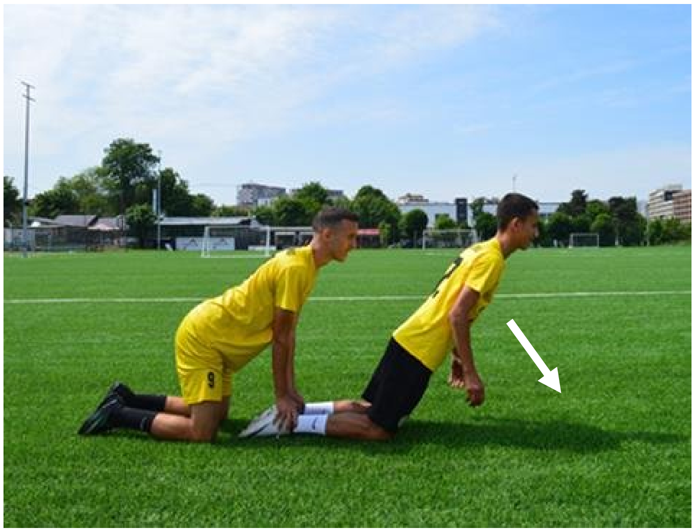

Level 4

Action: same as level 1.

Repetition: 2x 6-8 repetitions.

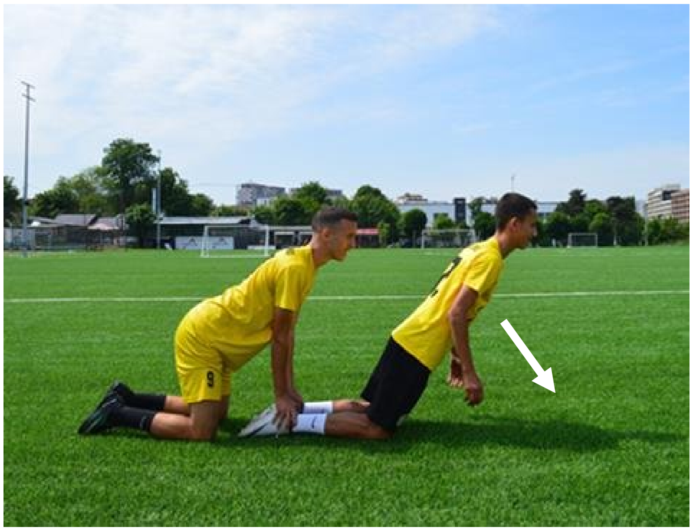

Level 5

Action: same as level 1.

Repetition: 2x 8-10 repetitions.

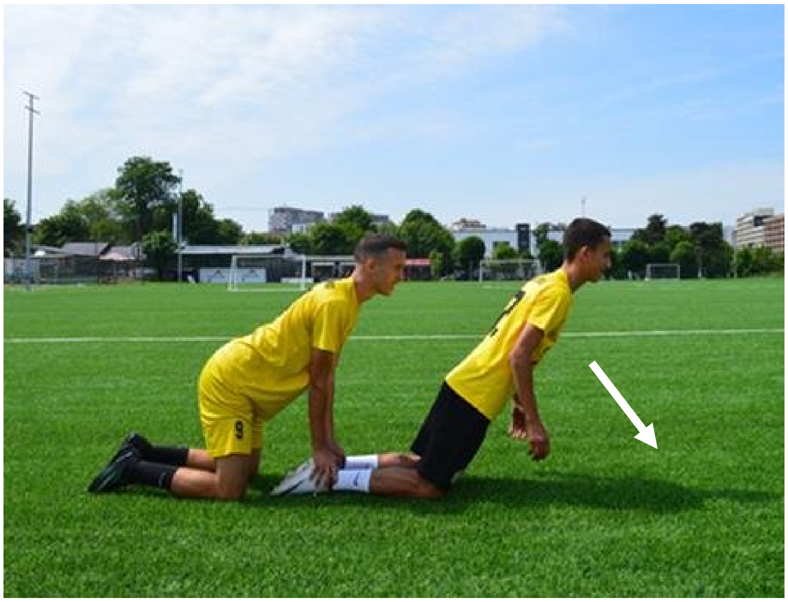

b. Hamstring walk-outs

Correct exercise posture:

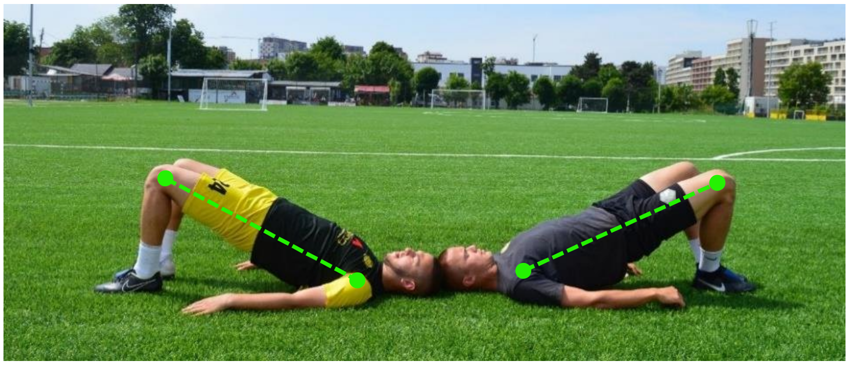

✓ Knees bent, and feet flat on the floor under the knees, shoulder width apart.

✓ Raise the hips to create a straight line from your knees to the shoulders.

Make sure to correct these mistakes:

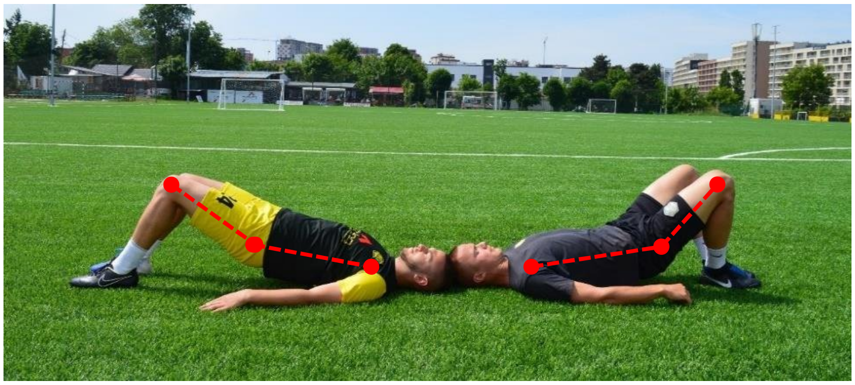

× Do **not** drop the hips.

Starting position: players are in pairs, lying on their backs. Their heads are placed close to each other. Players are facing away from each other. They are in a bridge position. The feet are planted.

Equipment needed: 1 ball per pair.

Level 1

Action: players walk forwards and backwards on their feet.

Explanation: *walk forward until the legs are fully extended and the weight is supported on the heels.*

Repetition: 2x 30 seconds.

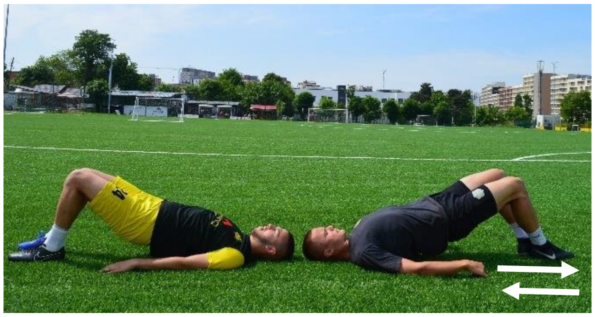

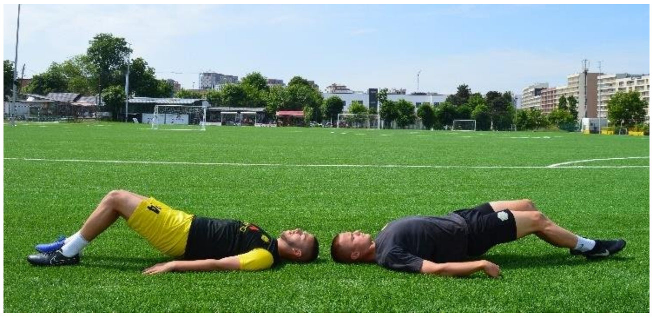

Level 2

Starting position: same as level 1. There is one ball per pair.

Action: players walk forwards and backwards on their feet. While walking forward and backward, they hand the ball back and forth overhead to their partner using their hands.

Repetition: 2x 30 seconds.

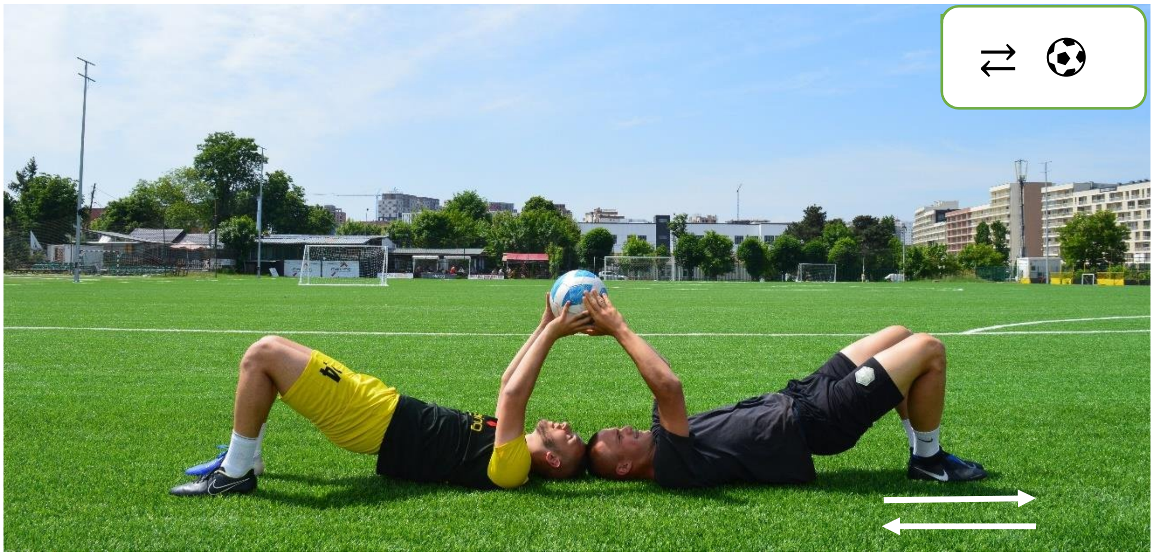

Level 3

Action: coach instructs a three-digit number. While walking forward and backward, if the last number is odd or even number (known previously for the players), they hand the ball back and forth overhead to their partner using their hands, e.g., *"odd" =pass the ball, "even" =keep the ball.*

Repetition: 2x 30 seconds.

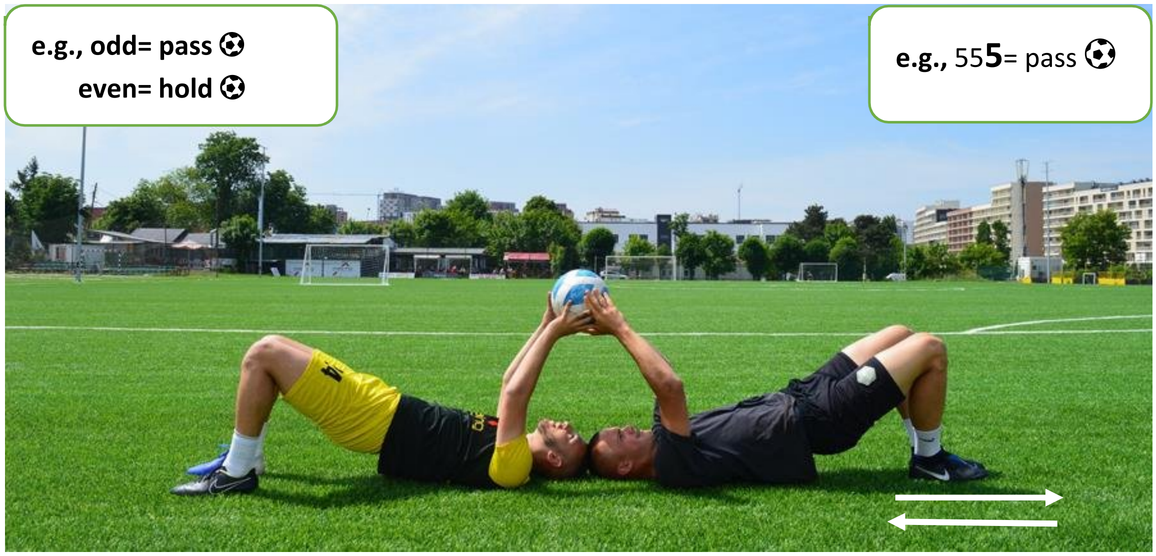

Level 4

Action: while walking forward and backward, they hand the ball back and forth overhead to their partner using their hands, when the coach instructs a specific number known previously to the players (5 numbers)*, e.g., pass the ball only when the instruction is 1,13,24,71,99 …*

Repetition: 3x 30 seconds.

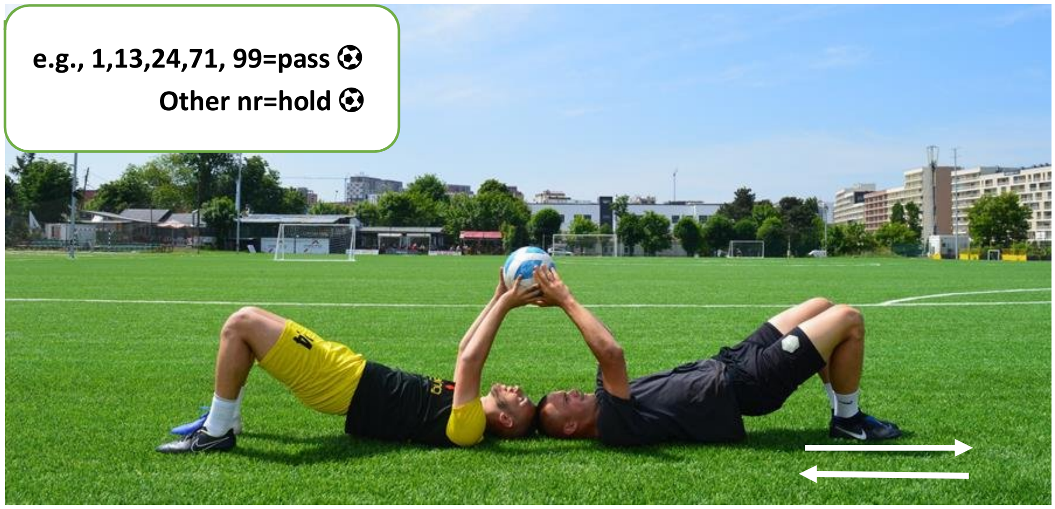

Level 5

Action: coach uses a calculation to have the odd and even numbers. While walking forward and backward, they hand the ball back and forth overhead to their partner using their hands, when calculation gives an odd or even number (known previously for the players), e.g., *"odd" =pass the ball, "even" =keep the ball.*

Repetition: 3x 30 seconds.

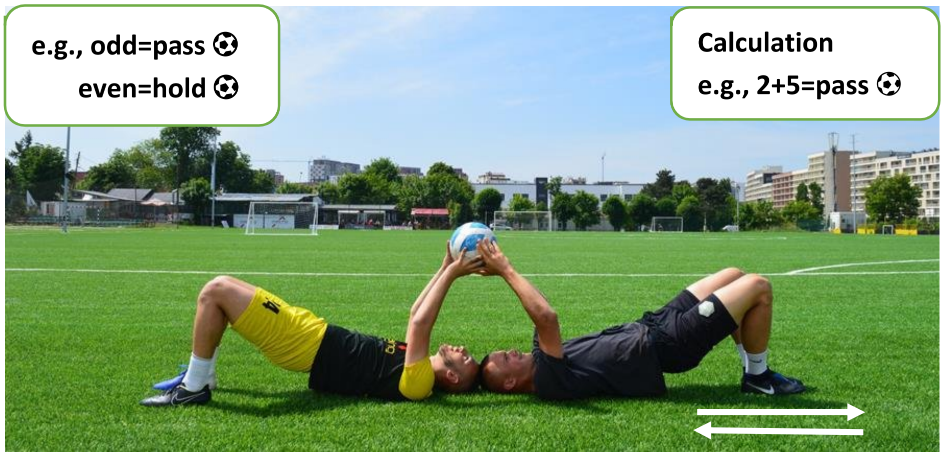

6.4. Gluteal muscle activation

a. Head, Shoulder, Hip, Knee, Ankle

Correct exercise posture:

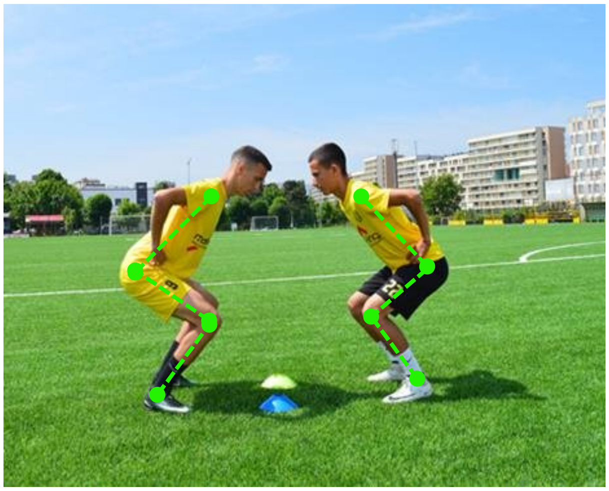

- ✓ In the front view, keep the hips, knees and feet in one line.
- ✓ Feet are shoulder width apart.
- ✓ The toes are facing forward.
- ✓ The knees should be only slightly in front of the ankle.
- ✓ Drive your hips back. Bend your hip and knee joints to 90° (squat position).
- ✓ Keep the back straight.
- ✓ Keep the feet planted (in the first three levels).

Make sure to correct these errors:

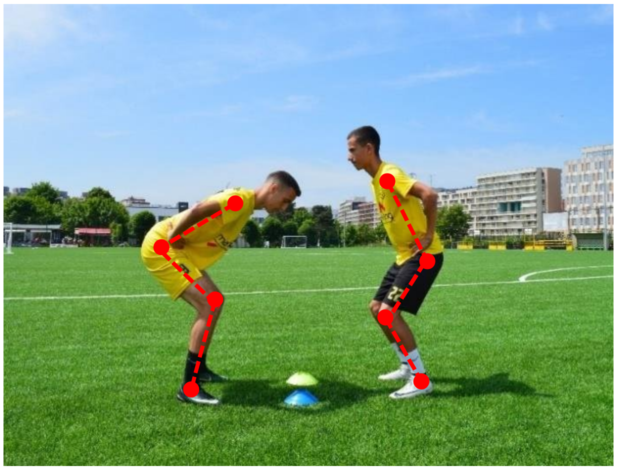

- × Do **not** let the knees move inwards.
- × No **not** round the back.

Starting position: Players are in 2 long rows facing each other in the 90° squat position. They face each other about 2 arm lengths apart. The ball (or later 2 cones) lie(s) midway between each pair. On instruction of the coach players subsequently touch various parts of their own body with both hands. On a specific instruction of the coach, e.g., 'ball', players compete about catching the object on the ground first (=winner). After each catch players change their partner, *e.g., losers, move one place to the left, winners moving one place to the right.*

Equipment needed: 1 ball and 2 cones of different colour per pair.

Level 1

Action: coach randomly instructs the body parts: head, shoulders, hips, knees, ankles. When the instruction is 'BALL', players compete to catch it first.

Repetition: 2x 6 repetitions.

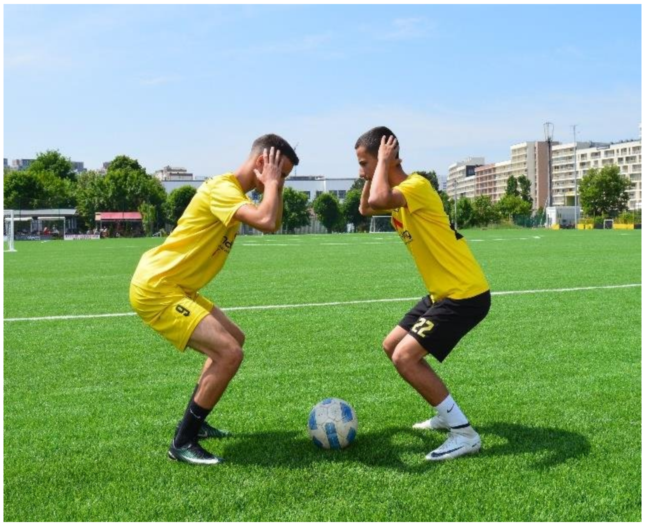

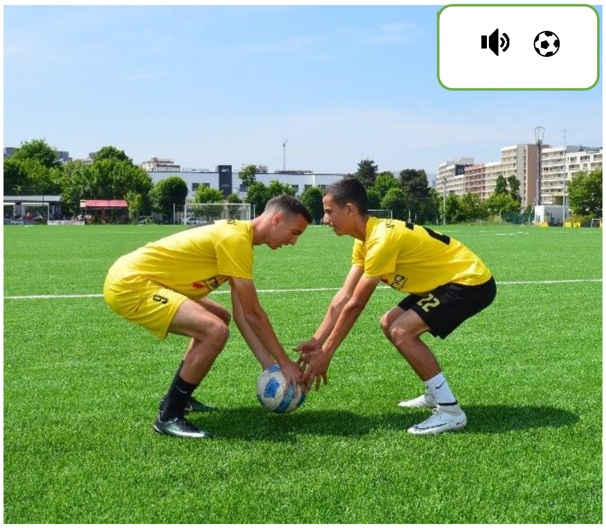

Level 2

Starting position: same as level 1. The ball is replaced with two cones of different colours.

Action: when the instruction is a colour, players compete to catch that cone first.

Repetition: 2x 6 repetitions.

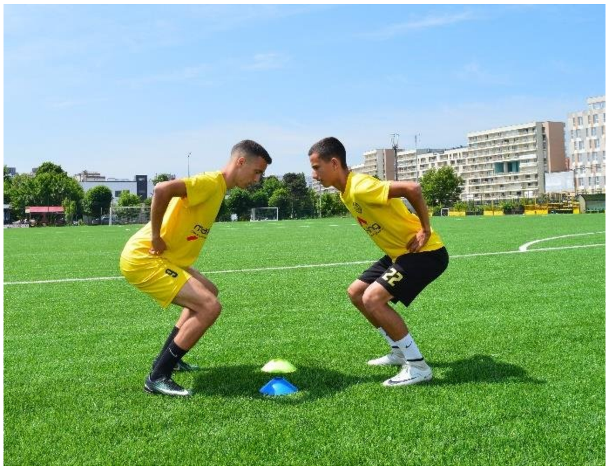
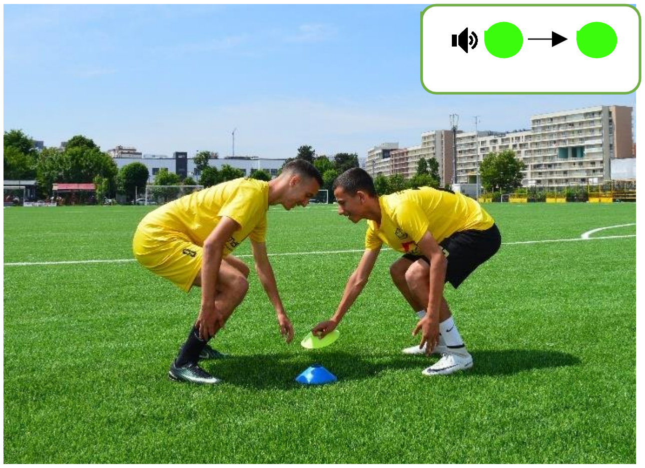

Level 3

Action: same as level 2. Players compete for the cone that was not instructed.

Repetition: 2x 8 repetitions.

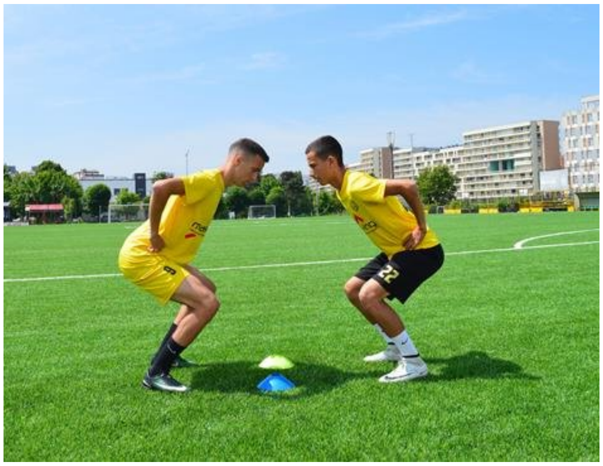
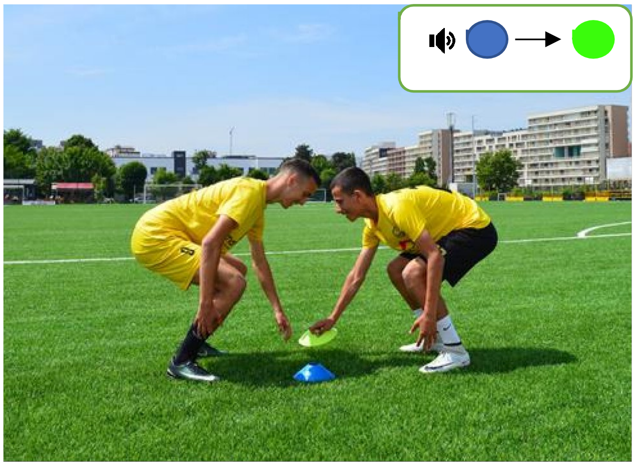

Level 4

Staring position: players tap the foot on the ground as fast as possible (with the toes moving up and down).

Action: coach replaces the colour of the cones for odd and even numbers., e.g., *"blue" =even and "green" =odd.* If the instruction is an odd number, players compete for the cone representing odd numbers.

Repetition: 2x 8 repetitions.

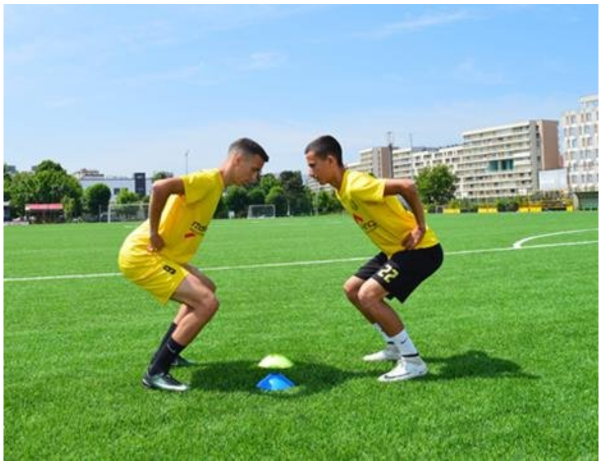
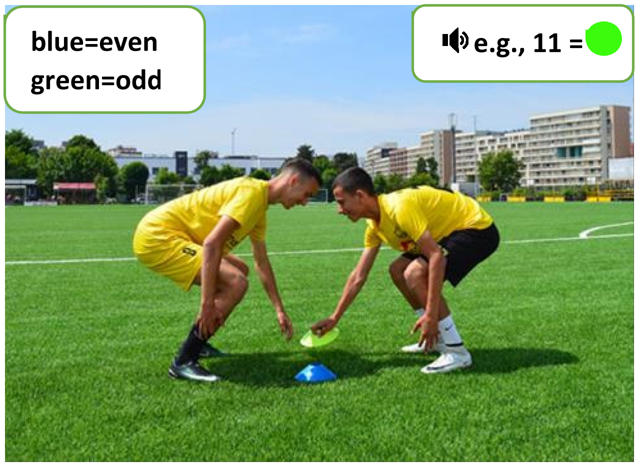

Level 5

Action: players tap the foot on the ground as fast as possible.

Each colour of the cones means a specific number told previously to the players, e.g., *"green" =4, and "blue" =7.* Coach instructs a three-digit number. Based on the last number of the instruction players compete for the cone represented by that number.

Repetition: 2x 10 repetitions.

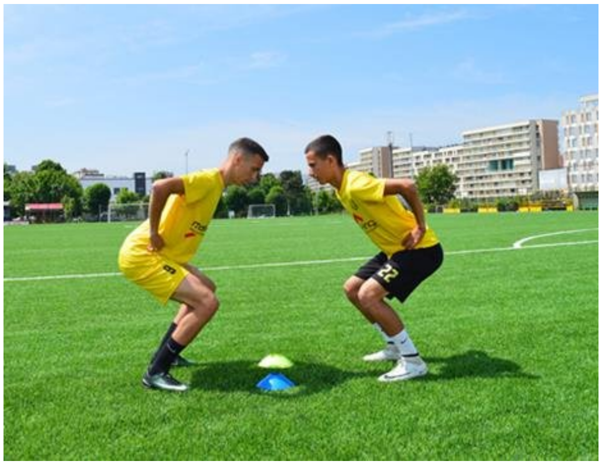
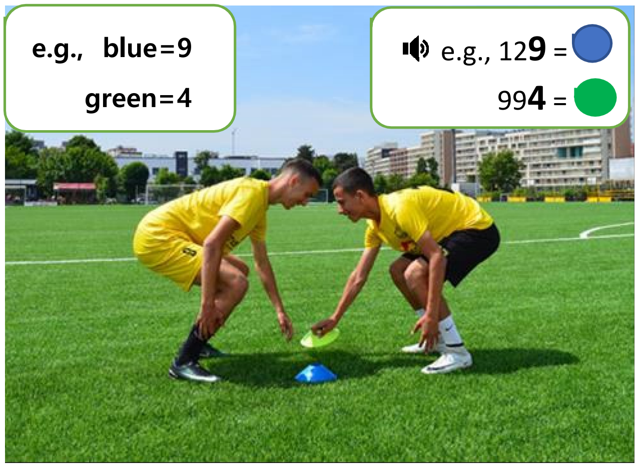

Level 6

Action: players tap the foot on the ground as fast as possible.

Players touch one level down from the instruction, e.g., *"head" means touching the "shoulder" ("shoulder"="hip", "hip"="knee", "knee"="ankle", "ankle"="head")*. Coach replaces the colours of the cones for odd and even numbers, e.g., *"blue"=even and "green"=odd.* If the instruction is an even number players compete for the cone representing even numbers.

Repetition: 2x 10 repetitions.

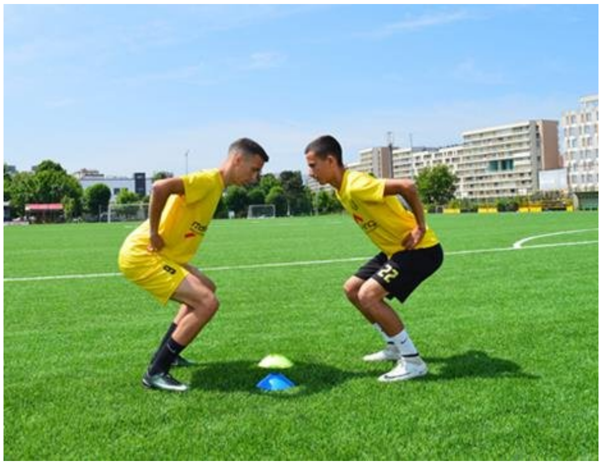

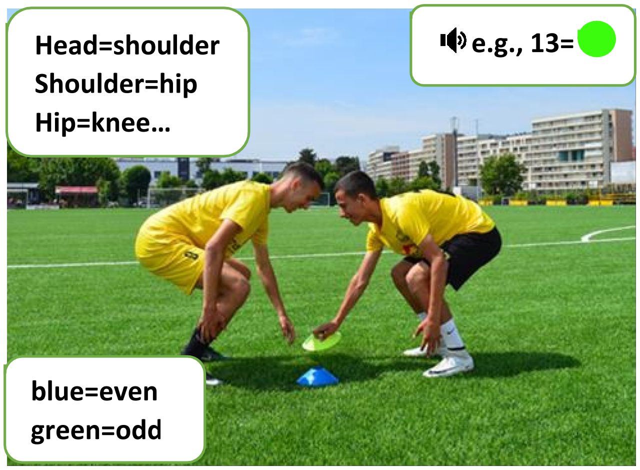

b. Squat Lunges

Correct exercise posture:

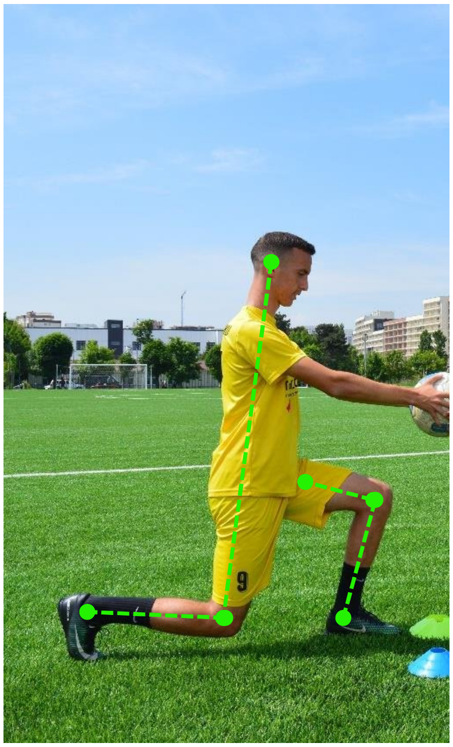

- ✓ Stand up straight and tall.
- ✓ The position of the back, neck and head should be in one line.
- ✓ Step forward with one foot until your leg reaches a 90-degree angle at hip and knee joint.
- ✓ Knee should be only slightly in front of the ankle.
- ✓ Keep the back straight.
- ✓ Keep the pelvis horizontal.

Make sure to correct these errors:

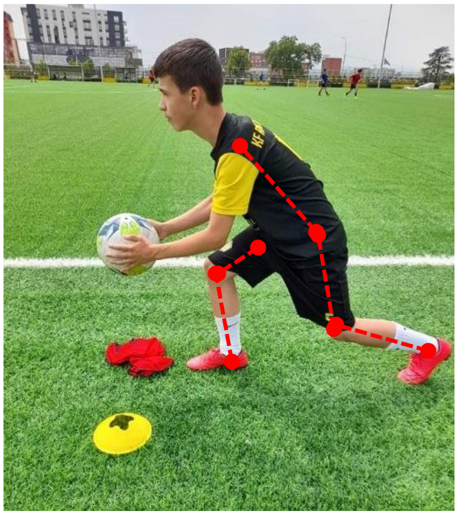

- × Do **not** touch the ground with the knee.
- × Do **not** let the knees move inwards.
- × Do **not** round the back.

Starting position: players are in pairs. They face each other about 2 arm lengths apart. In the middle of them are placed two cones of different colours. The distance between the cones is one hand width. Players rest their hands on the hips.

Equipment needed: 2 cones of different colours and 1 ball per pair.

Level 1

Action: coach instructs the colour. Players perform the lunge towards the cone with the respective colour.

Explanation: *if the colour of the cone placed on the right side of the player is instructed, the player performs the lunge with the right leg. Same applies for the other side.*

Repetition: 2x 8 repetitions (4 on each leg, random order).

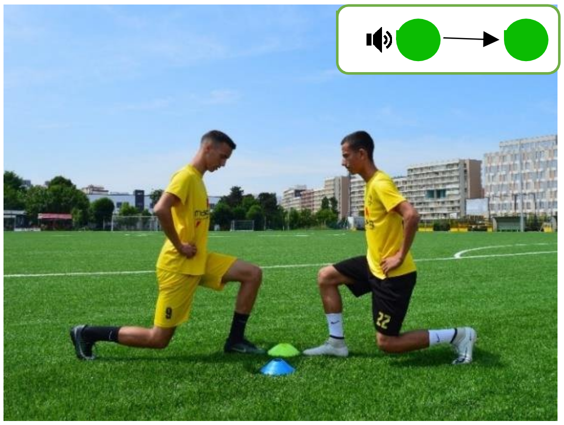

Level 2

Action: same as level 1. Players perform the lunge to the colour that was not instructed.

Repetition: 2x 10 repetitions (5 on each leg, random order).

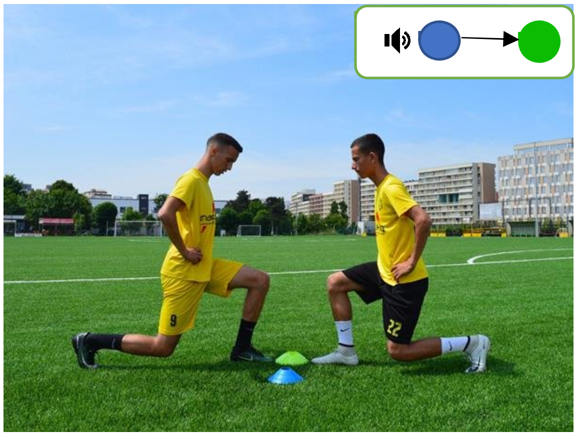

Level 3

Action: coach replaces the colour of the cones for odd and even numbers, e.g., *"blue" =even and "green" =odd.* If the instruction is an odd number players perform the lunge to the cone representing odd numbers.

Repetition: 2x 12 repetitions (6 on each leg, random order).

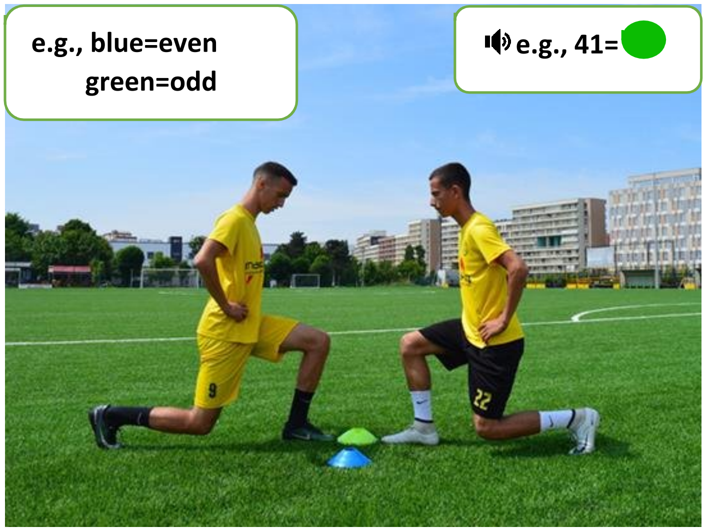

Level 4

Action: coach uses a calculation to have the odd and even numbers. Players perform the lunge to the cone representing these numbers, e.g., *"blue" =even and "green" =odd.*

Repetition: 3x 8 repetitions (4 on each leg, random order).

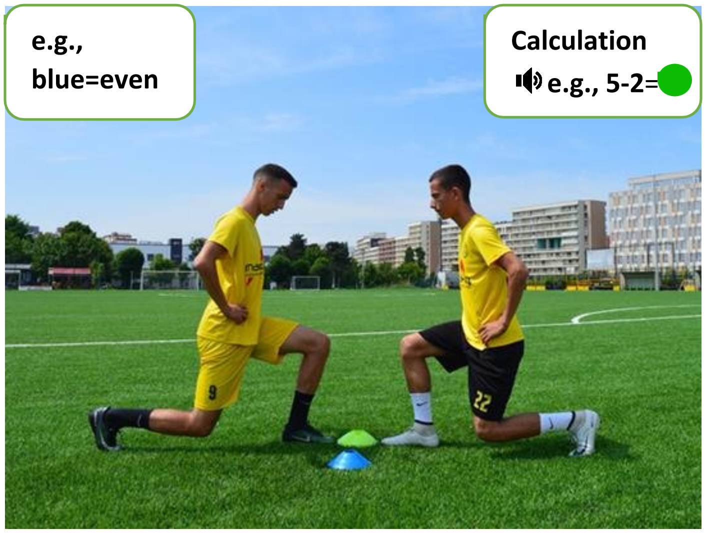

Level 5

Starting position: same as level 1. One ball per pair.

Action: same as level 4. They hand the ball to the partner when they go in a lunge position.

Repetition: 3x 10 repetitions (5 on each leg, random order).

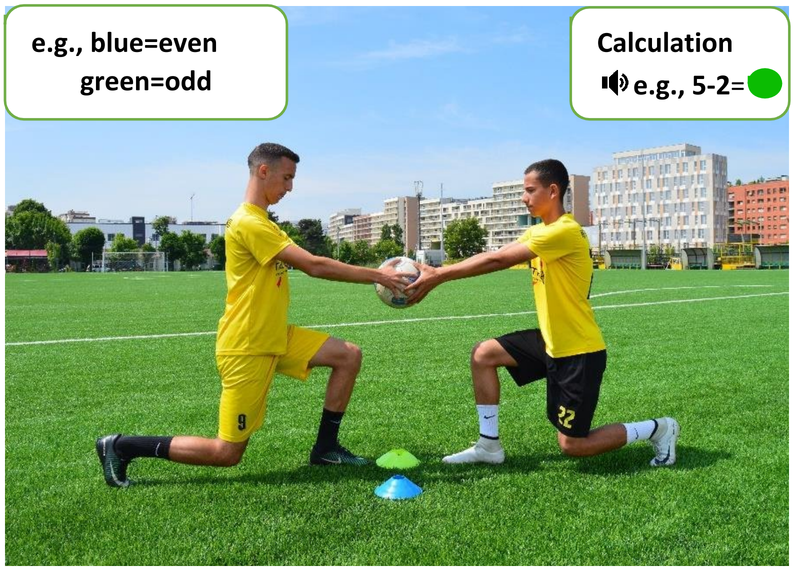

Level 6

Action: same as level 5. The eyes are closed.

Repetition: 3x 12 repetitions (6 on each leg, random order).

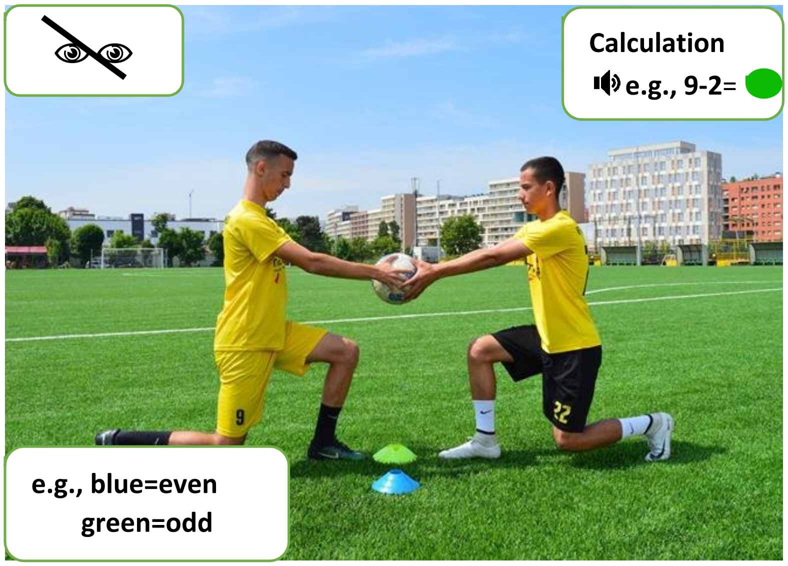

6.5. Plyometric exercises

a. Forward Jumps

Correct exercise posture:

- ✓ Squat down by bending knees and hips.
- ✓ Swinging arms back.
- ✓ Immediately jump forward while swinging arms forward and upward.
- ✓ Land on the balls of either both or one foot.
- ✓ Bend ankles, knees, and hips to absorb impact.

Make sure to correct these errors:

- × Do **not** let the knees move inwards.
- × Do **not** land with extended knees or on your heels.
- × Do **not** let your knees fall ahead of your toes.

Starting position: all players stand in one line next to each other approximately 1m apart.

Equipment needed: one ball per player.

Level 1

Action: players perform 3 forward jumps. They take off and land on both legs. They compete for the longest distance. Players can use the arms to gain movement. They walk back to the starting position to begin the next repetition.

Repetition: 4x 3 jumps.

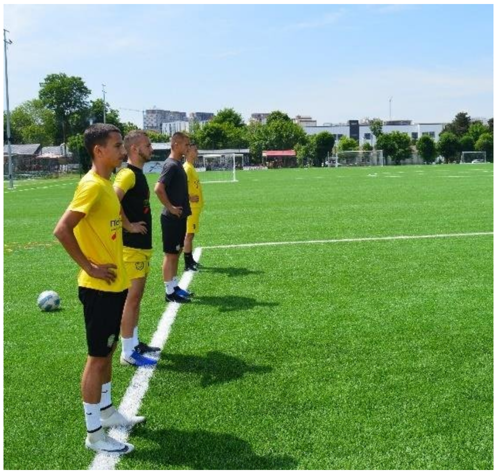

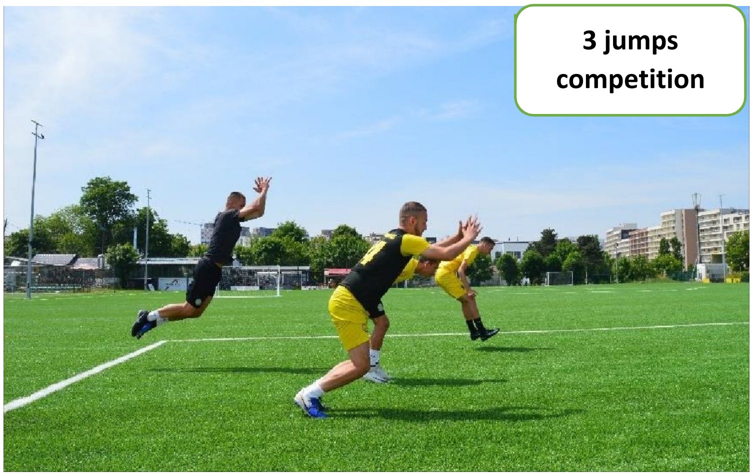

3 jumps competition

Level 2

Action: same as level 1. They take off on one leg, landing on both legs. They immediately remove one leg of the ground to perform the next jump (same applies for three jumps). They walk back to the starting position to begin the next repetition.

Repetition: 2x 3 jumps (on each leg).

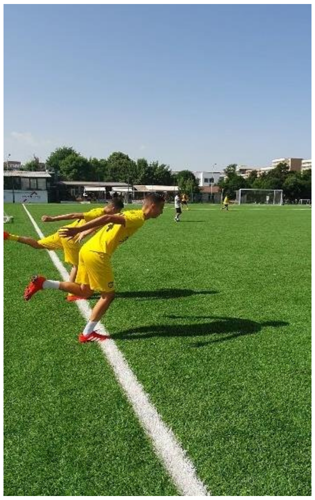

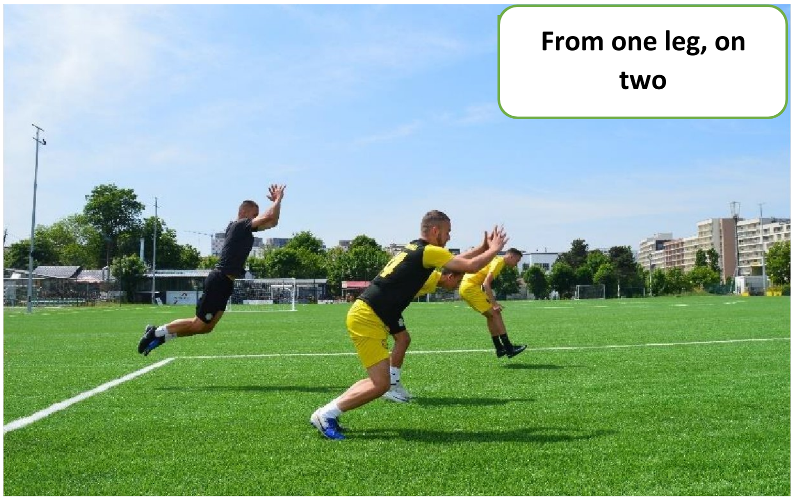

Level 3

Action: same as level 1. They take off on both legs, landing on one leg. They immediately turn their foot on the ground to perform the next jump (same applies for three jumps). They walk back to the starting position to begin the next repetition.

Repetition: 2x 3 jumps (on each leg).

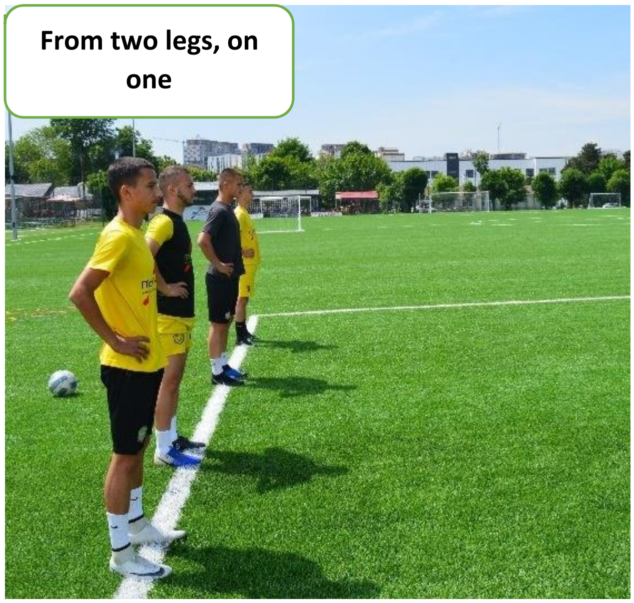

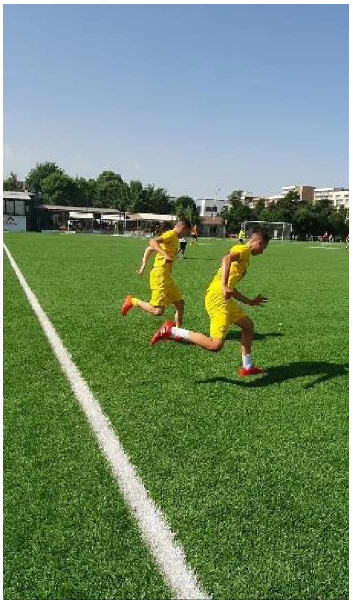

Level 4

Action: same as level 1. They take off on one leg, landing on opposite leg, **e.g.,** ***take off with the right leg, land on the left*** **(for three jumps).** They walk back to the starting position to begin the next repetition.

Repetition: 2x 3 jumps (on each leg).

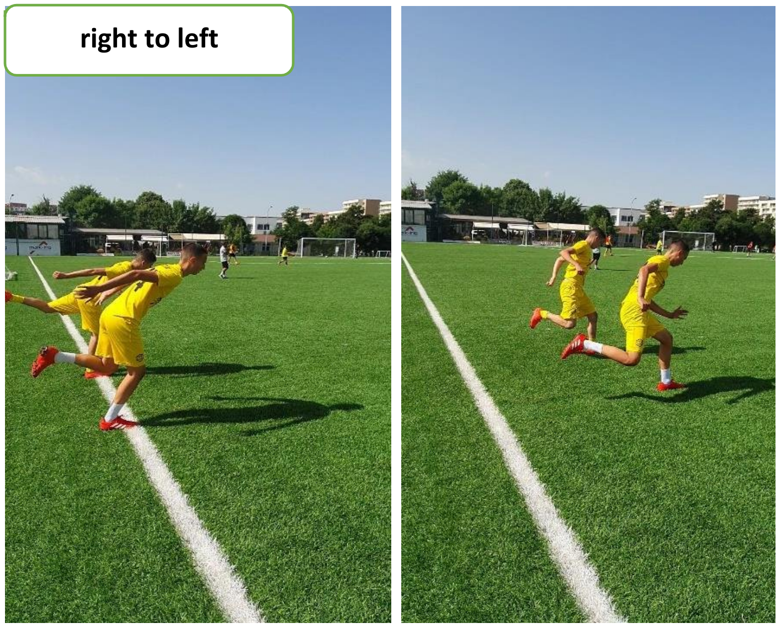

Level 5

Action: same as level 1. Players have the ball in their hands. They take off and land on the same leg **e.g.,** ***take off with the right leg, land on the right*** **(for three jumps).** They walk back to the starting position to begin the next repetition.

Repetition: 2x 3 jumps (on each leg).

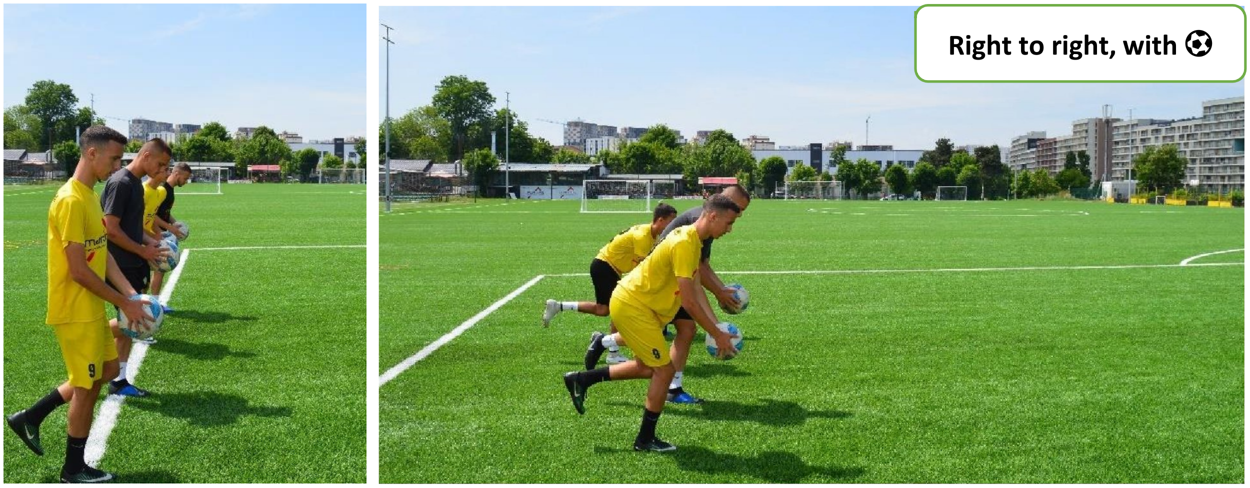

b. Skater Jumps

Correct exercise posture:

- ✓ Start with feet hip width apart and knees slightly bent.
- ✓ Push off your right foot to hop to your left and vice versa.
- ✓ Bend ankles, knees, and hips to absorb impact.

Make sure to correct these errors:

- × Do **not** let the knees move inwards.
- × Do **not** land with extended knees or on your heels.
- × Do **not** let your knees fall ahead of your toes.

Starting position: 2 cones placed 1.25m apart are (for lateral jumps) the starting point of this exercise with the player standing between them. 7 cones at 1m distance diagonally, and approx. 1m width apart are placed for skater jump exercises. Behind the cones 3 hurdles (or other objects) of 15cm height are placed, at approx. 40 cm distance. Prepare 3-4 courses depending on team size (max 6 players per course).

Equipment needed: 9 cones, 3 hurdles and 2 balls for each course.

Level 1

Action: player performs 6 lateral jumps between the first two cones at 'start line'. Player moves on to perform the skater jumps diagonally and finally jumps over the hurdles with two legs. The player jogs back to the starting position. Next player starts when the previous player finishes skater jumps.

Repetition: 4 repetitions.

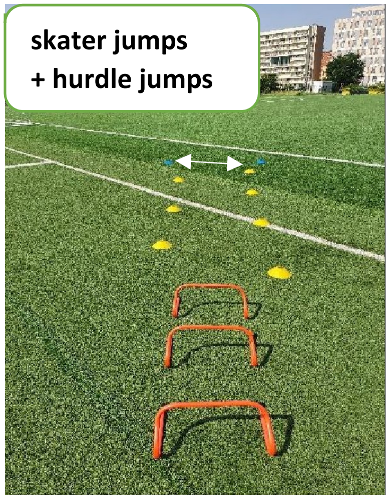

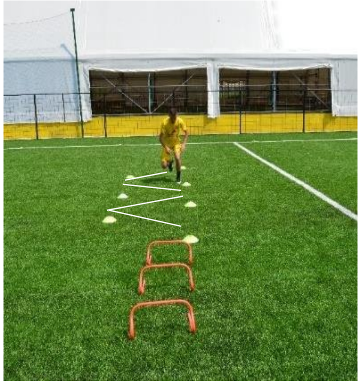

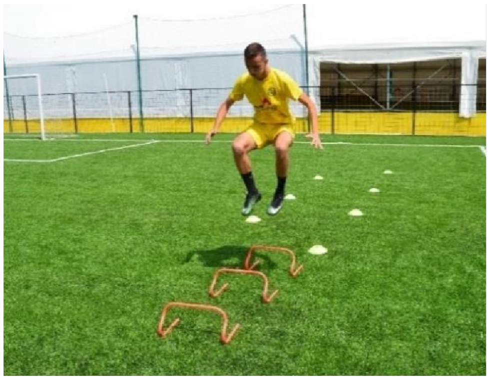

Level 2

Action: same as level 1. The player jumps over the hurdles on one leg. The leg remains the same.

Repetition: 4 repetitions (2 on each leg).

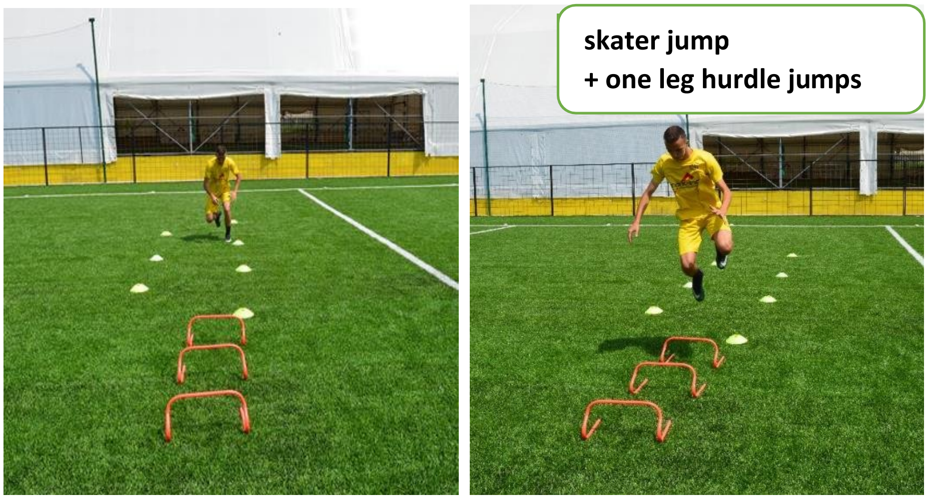

Level 3

Starting position: cone width distance for lateral jumps at the starting line is increased to 1.5m. Cone distance for skater jumps is increased to 1.25m (diagonally). Each course/station has one ball.

Action: same as level 2. Players additionally take the ball in their hands. Players jog to the starting point to hand the ball to the next player.

Explanation: *in order to save time, use more balls, so that the next player can start if the previous player finishes their skater jumps.*

Repetition: 4 repetitions (2 on each leg).

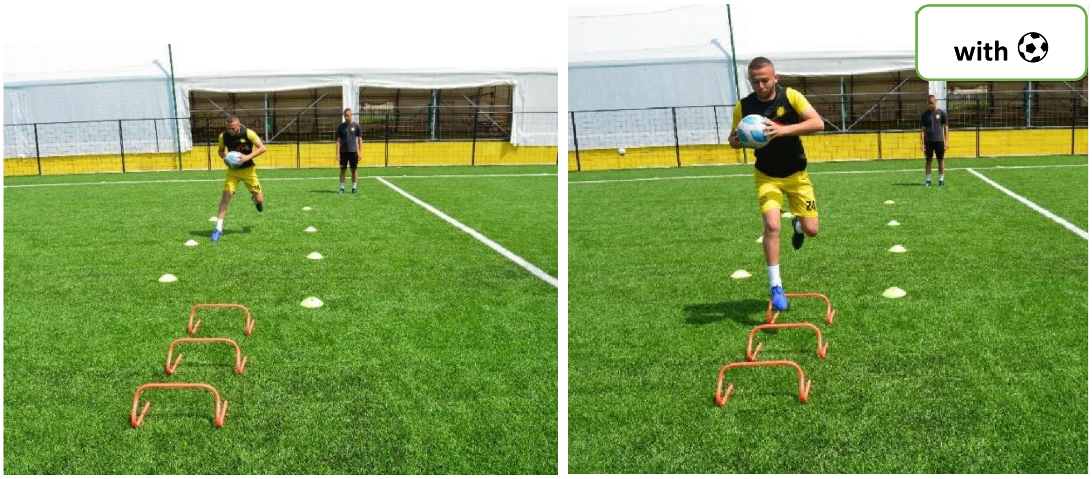

Level 4

Action: same as level 3. The player completes the course as fast as possible and hands the ball to the next in line who does the same. Teams of the different courses compete for the fastest finish. One competition finishes when all players of a course/station have finished one cycle (repetition).

Explanation: *number of players per team is set by the coach (max 6 players per team).*

Repetition: 4 repetitions (2 on each leg).

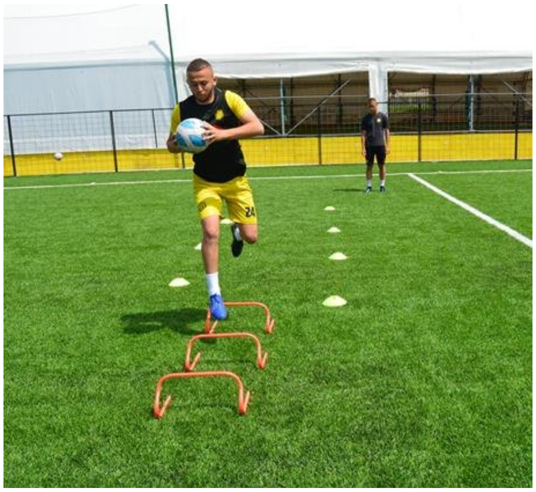

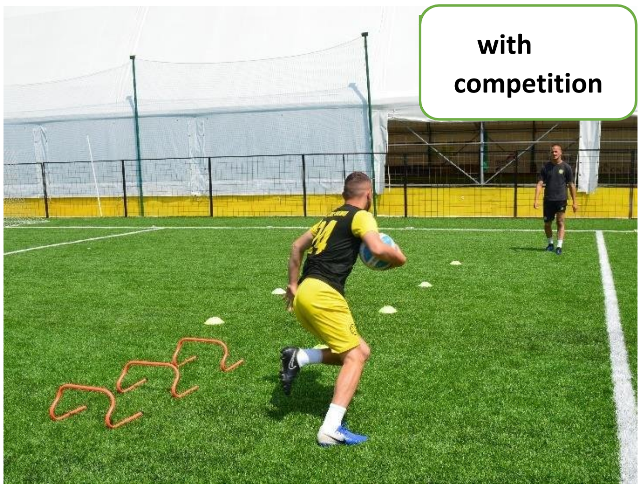

Level 5

Starting position: there are two balls per course.

Action: same as level 4. Players carry 2 balls, one in each hand.

Repetition: 4 repetitions (2 on each leg).

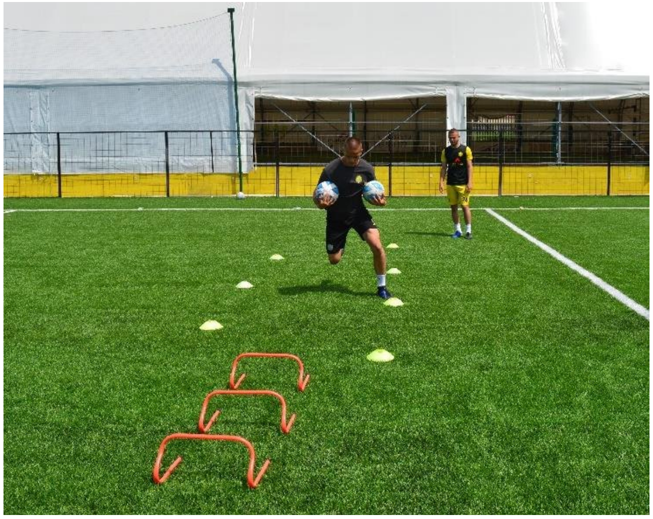

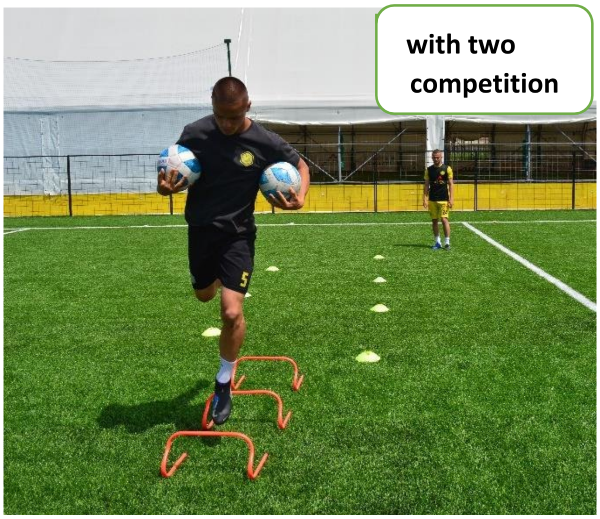

5.6. Running/Sprinting exercises

- ✓ Keep your upper body straight.
- ✓ Hips, knees and feet should be aligned.
- ✓ Keep your pelvis horizontal.
- ✓ Swing your arms sideways to the body (**not** across the body).

Make sure to correct these errors:

- × Do **not** let the legs cross the midline.
- × Do **not** let the knees move inwards.

a. Diagonal running

Starting position: coach and players stay 10m apart facing each other. Players stay in pairs. Coach stands in the middle of 2 cones of different colour, placed 10m apart. Another cone (exact colour does not matter) is placed between the coach and the players. Players in pair jog (medium jog speed) next to each other straight forward to the cone placed in front of them. Coach instructs a command (verbal or visual) just before players reach the cone and players run with high speed diagonally to one of the cones located on the side of the coach, competing to reach the cone first.

Equipment needed: 5 cones and two balls for each course.

Level 1

Action: the coach provides verbal instruction of one cone's colour, e.g., "red" or "blue". Players continue to run to the instructed cone, competing to reach the cone first.

Remark: *Without a proper warm-up **avoid** competition. If there is no competition, players should increase their running speed with each repetition.*

Repetition: 3 repetitions per pair.

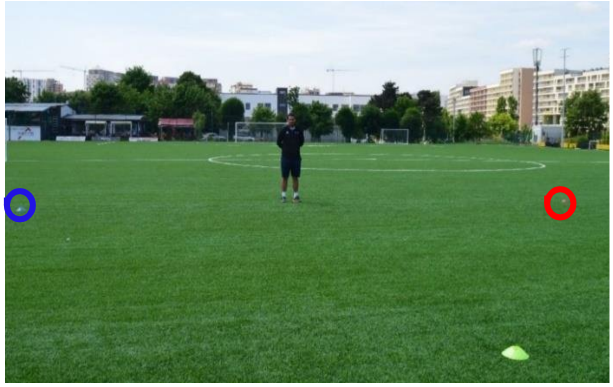

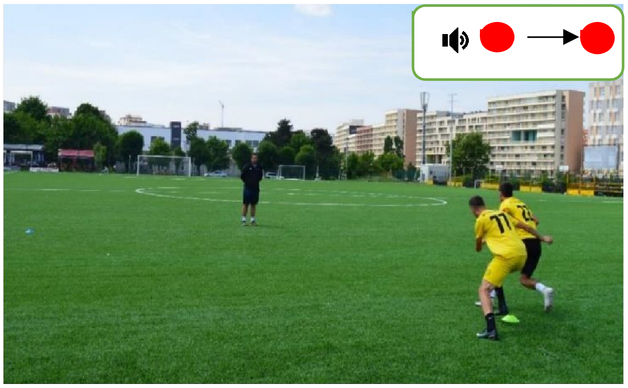

Level 2

Action: same as level 1. Players run to the cone that was not instructed.

Repetition: 3 repetitions per pair.

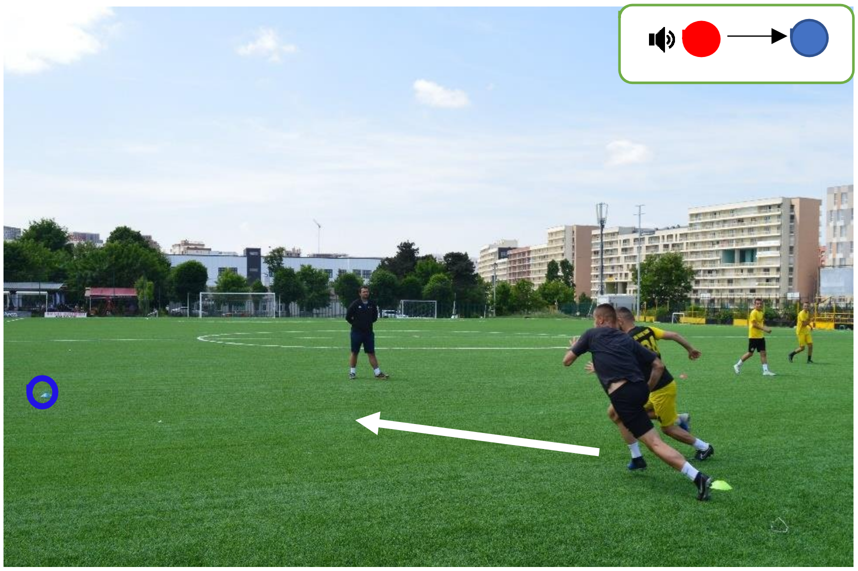

Level 3

Starting position: coach holds 2 different coloured cones in his hands behind his back. The colours are matched by the cones on the ground.

Action: coach provides a visual instruction. He shows the players one of the cones. Players run to the same colour of the cone on the ground.

Repetition: 3 repetitions per pair.

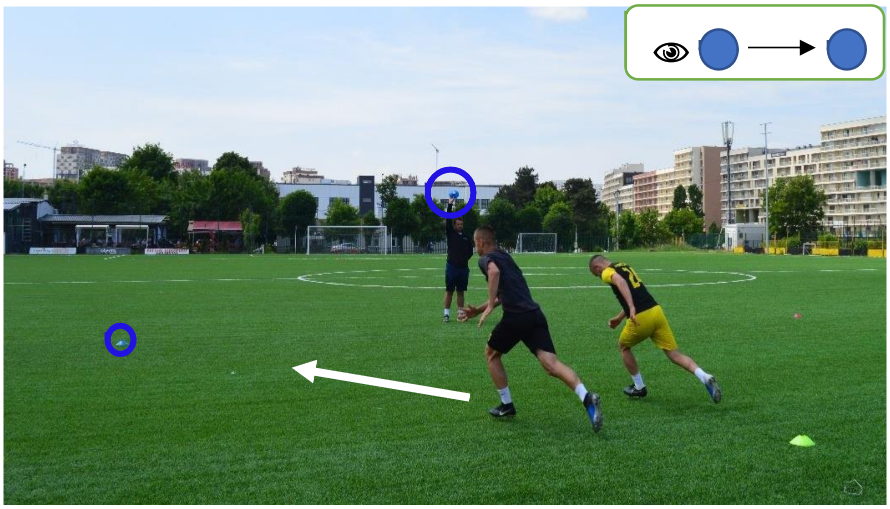

Level 4

Action: same as level 3. Players run to the cone that was not instructed.

Repetition: 3 repetitions per pair.

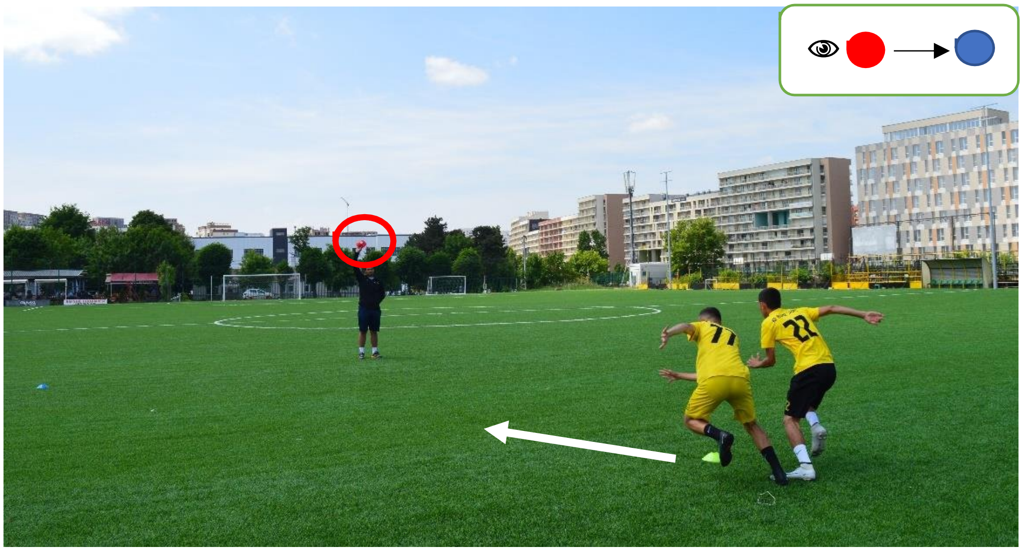

Level 5

Action: coach provides a verbal instruction. He replaces the colour of the cones for odd or even numbers, *e.g., "blue"=odd, "red"=even.* Players run to the cone representing those numbers.

Repetition: 3 repetitions per pair.

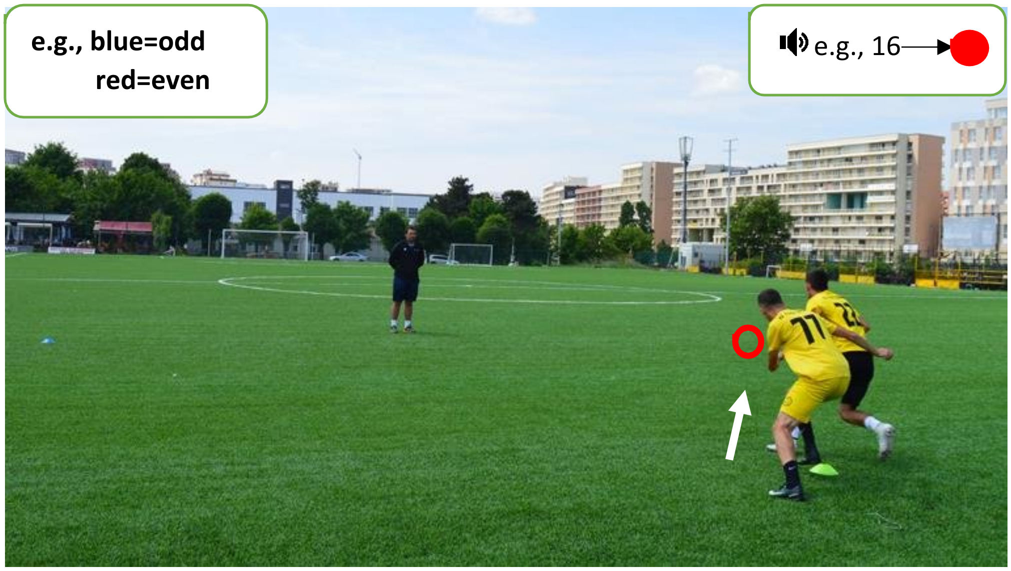

Level 6

Starting position: each player has a ball at their feet.

Action: the coach uses a calculation to have the odd and even numbers. Players dribble the ball to the cone representing those numbers.

Repetition: 3 repetitions per pair.

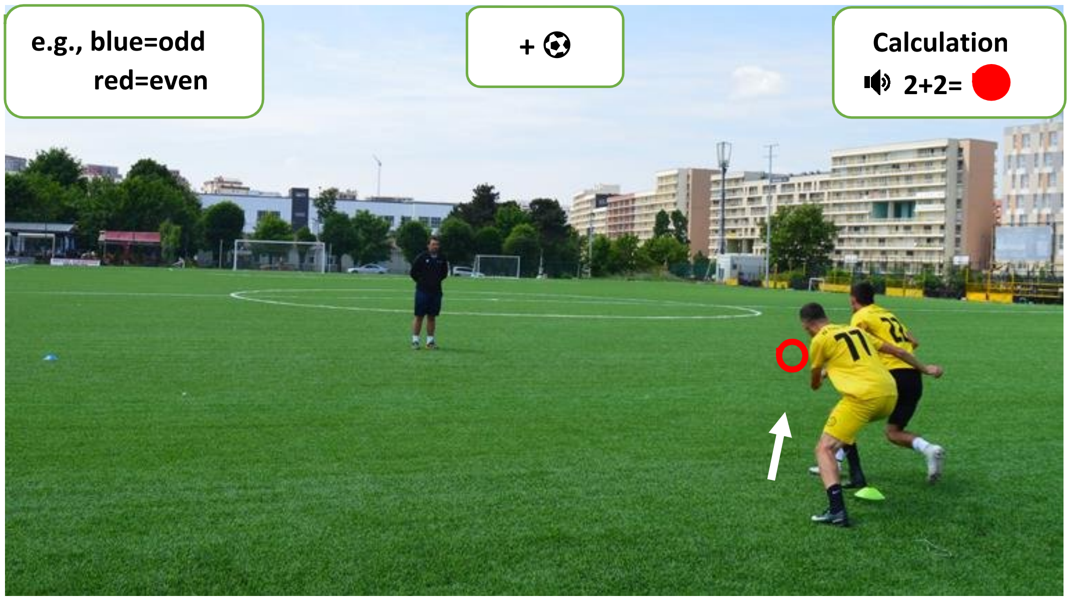

b. Forward running

Starting position: 2 cones with different colours are placed at a distance of 10m. Players are separated into 2 groups (max 6 players per group). They stand behind the first cone. Players in pairs of different teams start jogging and pushing each other side-to-side at shoulder level from the first cone until they reach about midway between the cones, when there is an instruction from the coach.

Equipment needed: 2 cones and two balls for each course.

Level 1

Action: when coach instructs "FORWARD" or "BACKWARD", players run forward to the cone placed in front ("forward") of them or behind ("backward") of them respectively as fast as they can, competing to reach the cone first.

Remark: *without a proper warm-up **avoid** competition. If there is no competition, players should increase their running speed with each repetition.*

Repetition: 3 repetitions per pair.

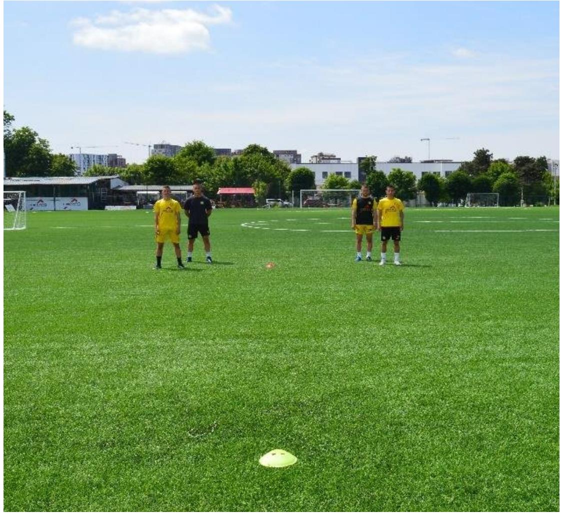

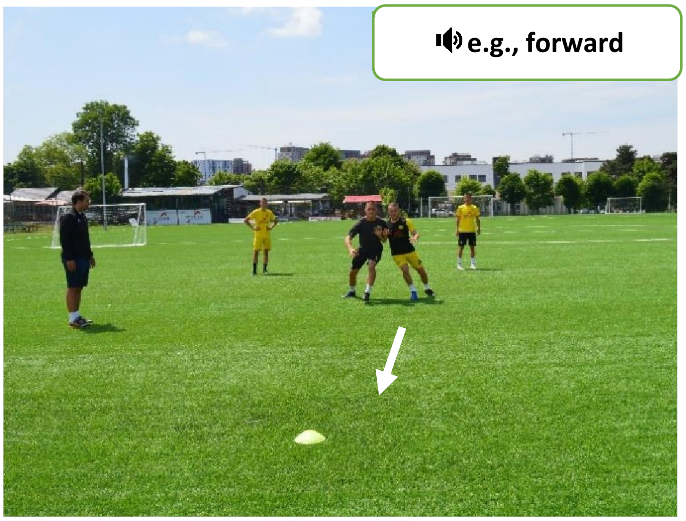

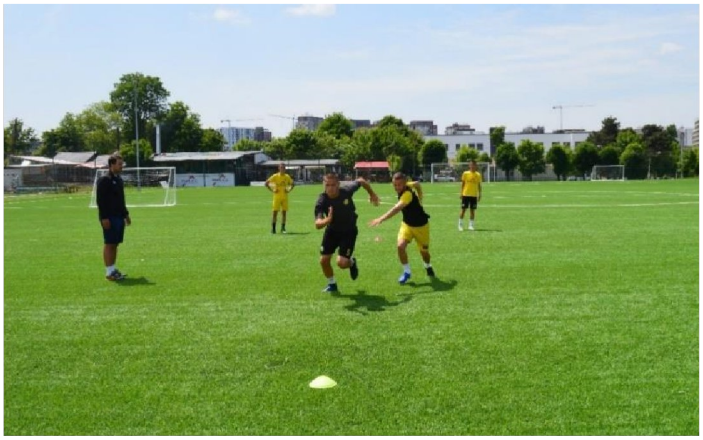

Level 2

Action: coach instructs one of the cone's colours, e.g., "red" or "green". Players run forward to the instructed cone.

Repetition: 3 repetitions per pair.

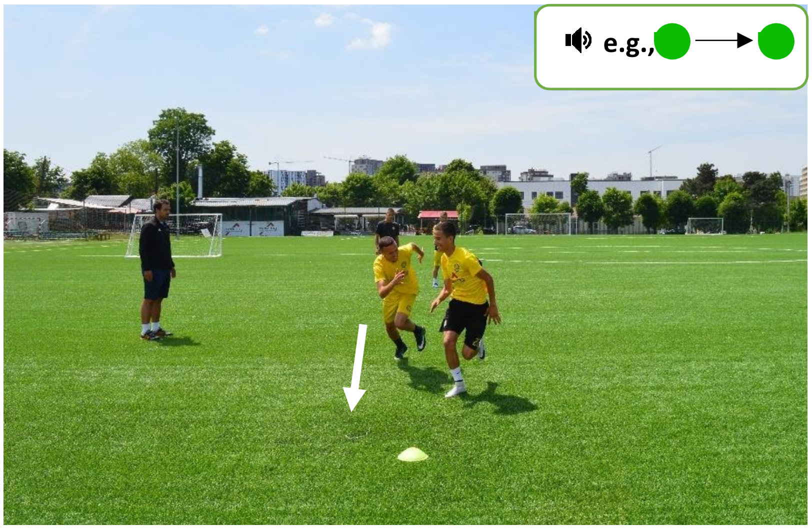

Level 3

Action: same as level 2. The players run forward to the cone that was not instructed.

Repetition: 3 repetitions per pair.

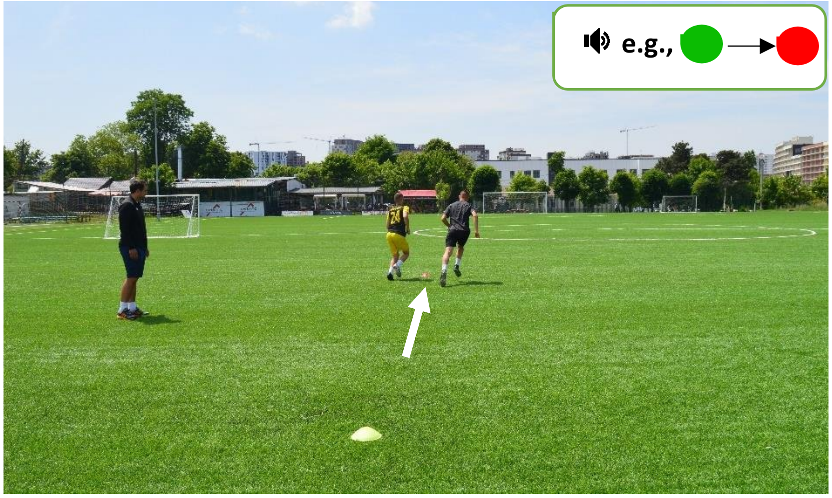

Level 4

Action: coach replaces cones' colours for odd or even numbers, *e.g., "green"=odd, "red"=even.* Coach instructs numbers. Players run forward to the cone representing those numbers.

Repetition: 3 repetitions per pair.

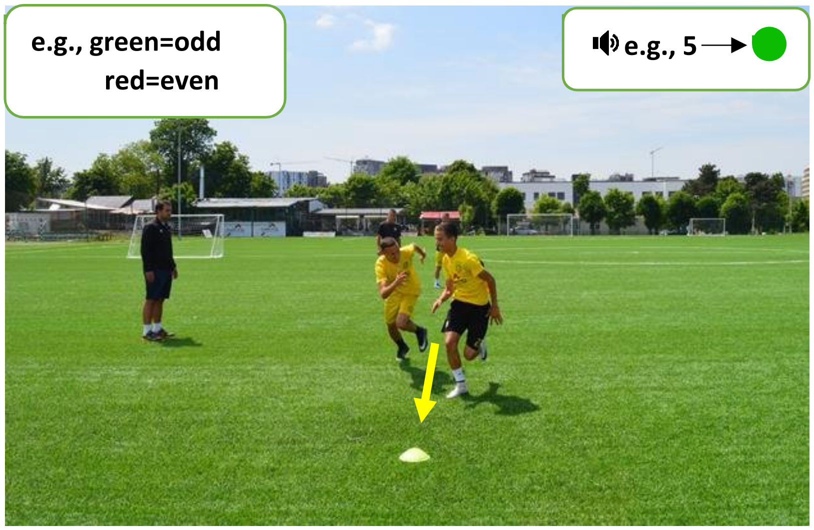

Level 5

Action: coach uses a calculation to have the odd and even numbers. Players run to the cone representing those numbers.

Repetition: 3 repetitions per pair.

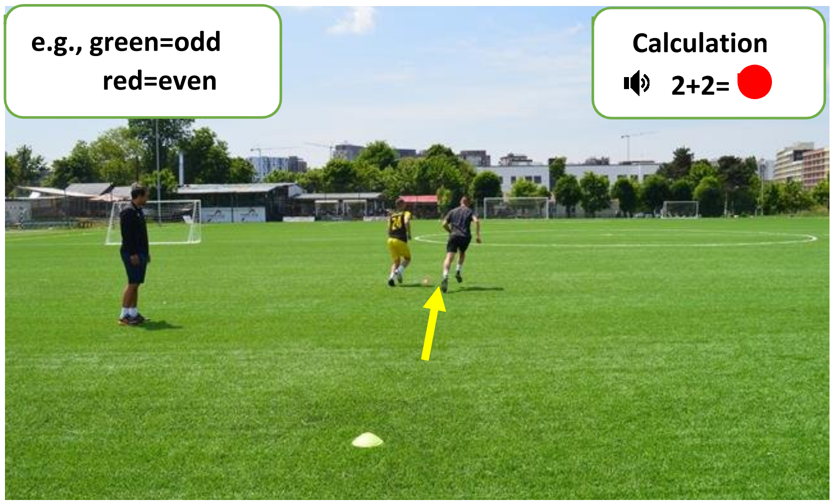

Level 6

Starting position: Each player has a ball at their feet.

Action: coach instructs a three-digit number. If the last number is odd players run to the cone representing odd numbers.

Repetition: 3 repetitions per pair.

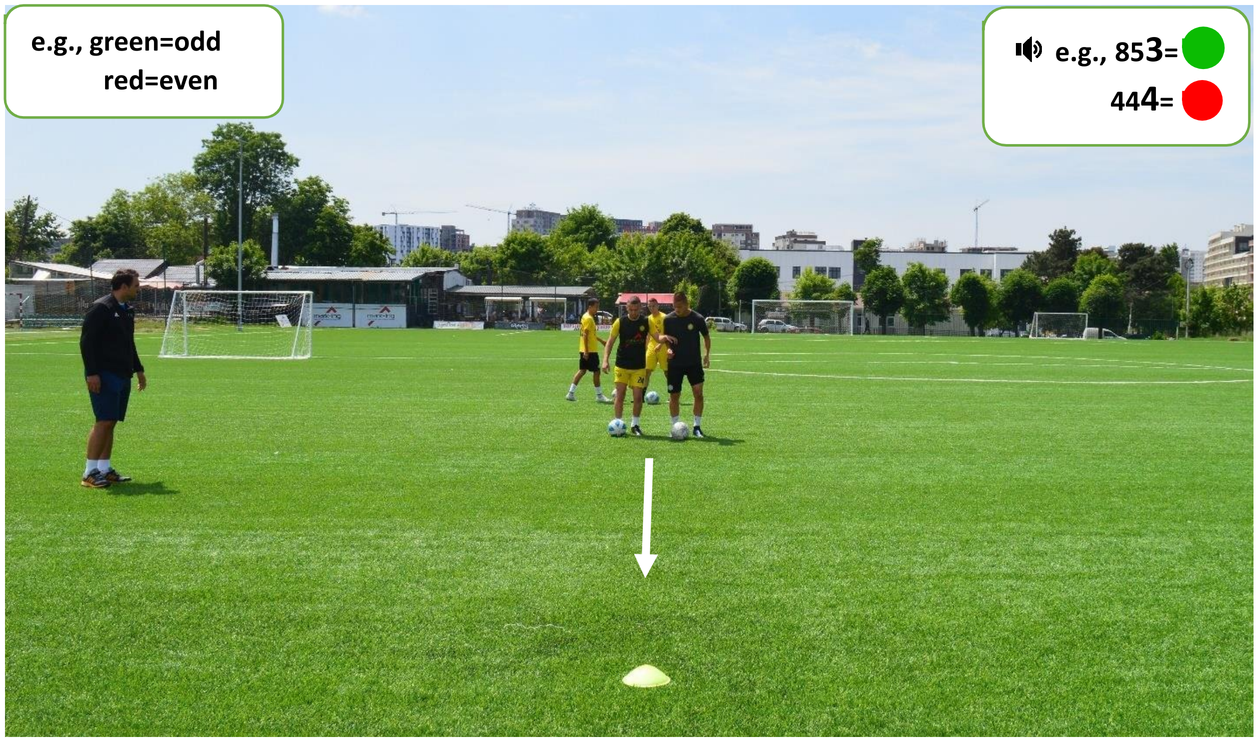

5.7. Games

a. Tic-Tac-Toe

Starting position: 9 hoops in 3 rows are placed in close proximity on the field to mark a tic-tac-toe field. Two cones are placed at a distance of 5m on opposite sites from this field represent the starting point of two teams. Teams consist of four players each. The first three players of each team have a training kit in their hand.

Equipment needed: 9 hoops, 2 cones (placed on the ground), and 6 training kits of two colours (one colour for each team).

Action: the first player in line from each team starts to run to the hoops and places the training kit in one of the hoops. Then they run back as quick as they can, return to the cone marking the starting point and provides a high five to the next player in line, who then starts towards the hoops. Aim is to create 3 kits in a row with your kits of your own colour. The 3 in a row can be diagonally, horizontally or vertically. If the game is not finished after the first three players, the fourth player starts moving one kit to an empty hoop set previously by his teammates, until the game ends.

Remark: players are not allowed to waste time at the hoops. They have to place/move the kit and return to their teammates as soon as possible.

Repetition: 3-5 games.

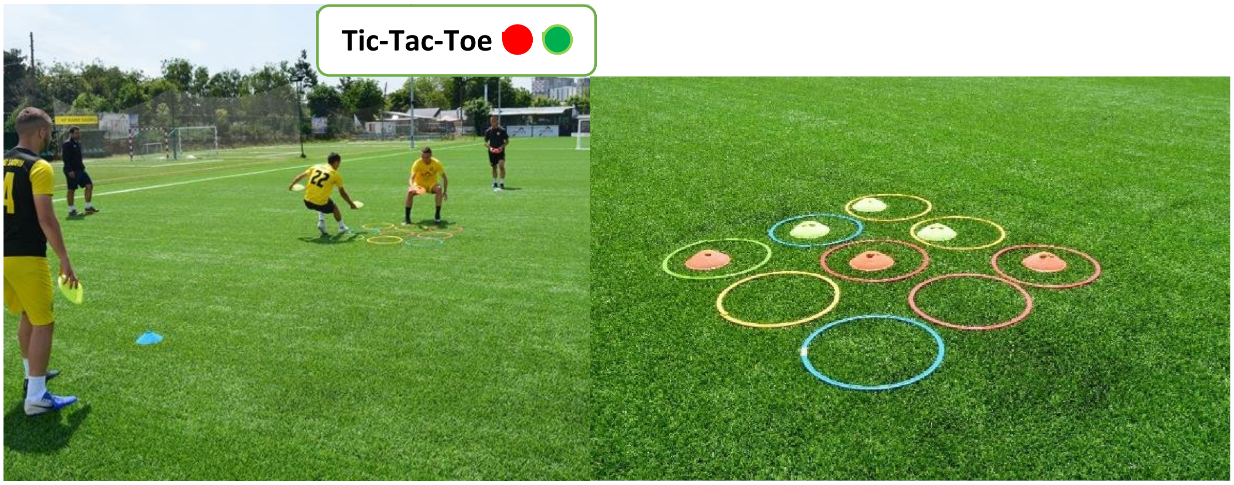

b. Header Game

Starting position: players are divided into 2 groups (max. 6 players per group). Both groups stand on the goal line 5 m away from either side of the post. The group that heads first, places one player (header) 5-8m into the penalty box line facing the other group members. The other group places one in the goal (role of the goalkeeper).

Equipment needed: balls, 5x2m goal (in case of its absence it can be modified, e.g., with cones or training kit).

Action: the player who heads first stands about 8m away from his teammates in the penalty box. Then the first mate of his team at the goal line throws a ball towards the header of his own team aiming for the 5m line. The header tries to score, which the other team tries to prevent by the allocated goalkeeper. The header then becomes the goalkeeper. The goalkeeper becomes the last in the line. Then the action repeats itself.

Repetition: 4 to 5 repetitions for each player.

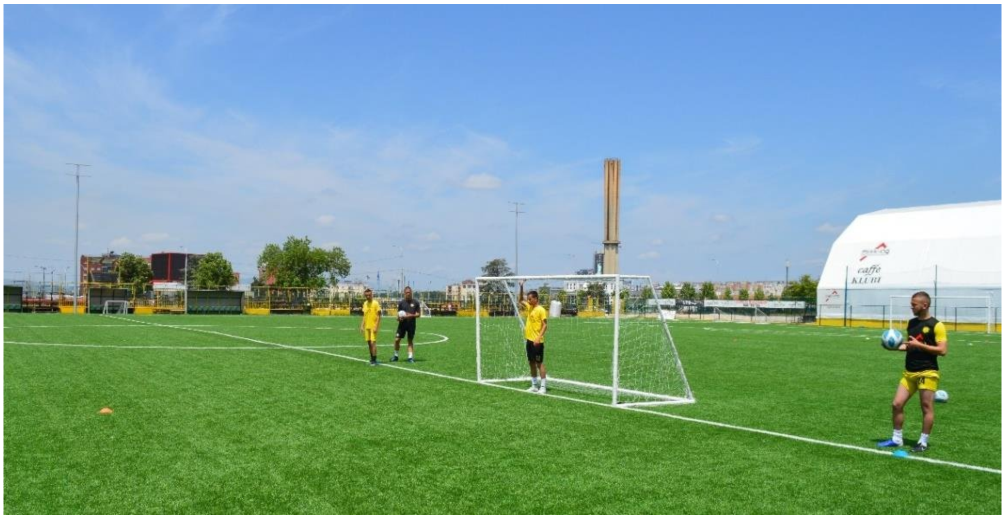

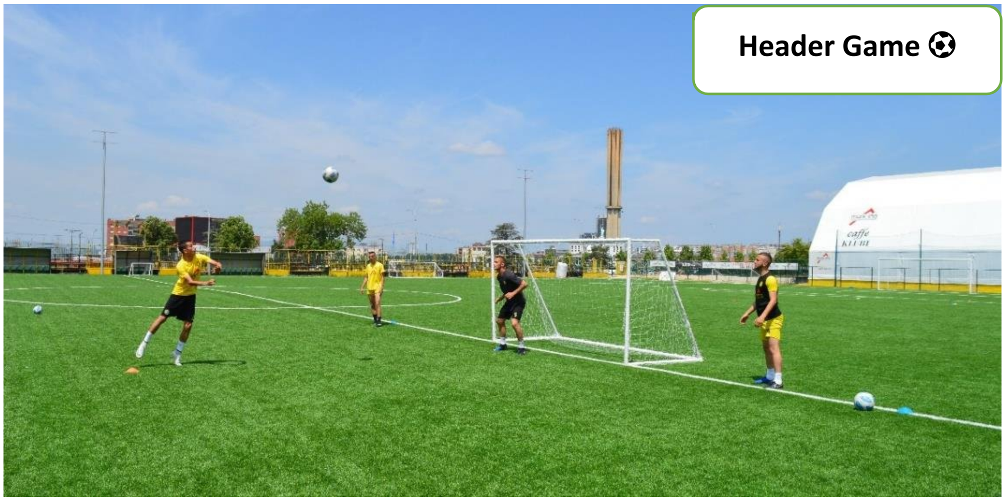

Header Game ⚽

c. Dribbling game

Starting position: There is a cone marking the starting point. At 3m distance from that cone 6 cones are placed in a row on the ground each 1m apart. Players are divided into teams of 4 players. They stand behind each other at the starting cone.

Equipment needed: 7 cones and one ball per team.

Action: the first player dribbles through the cones without touching the cones with either the ball or feet as fast as possible. The player then runs back to the first cone, gives the 2nd player a high five and hands the ball over to the 2nd player. The 2nd player performs the same action and so forth. Teams compete on who finishes the dribbling first.

Repetition: 3-5 games.

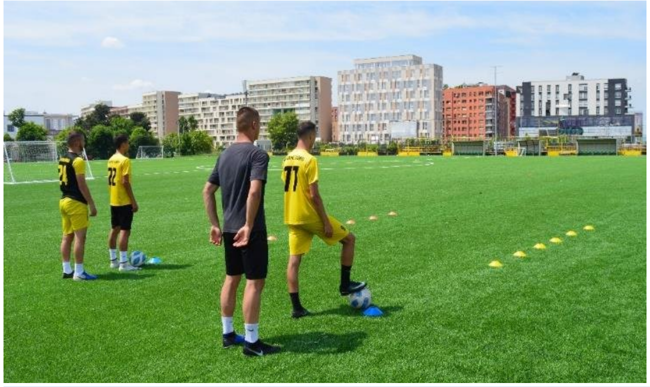

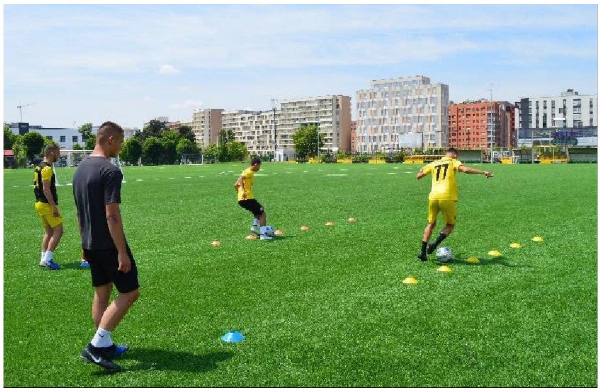

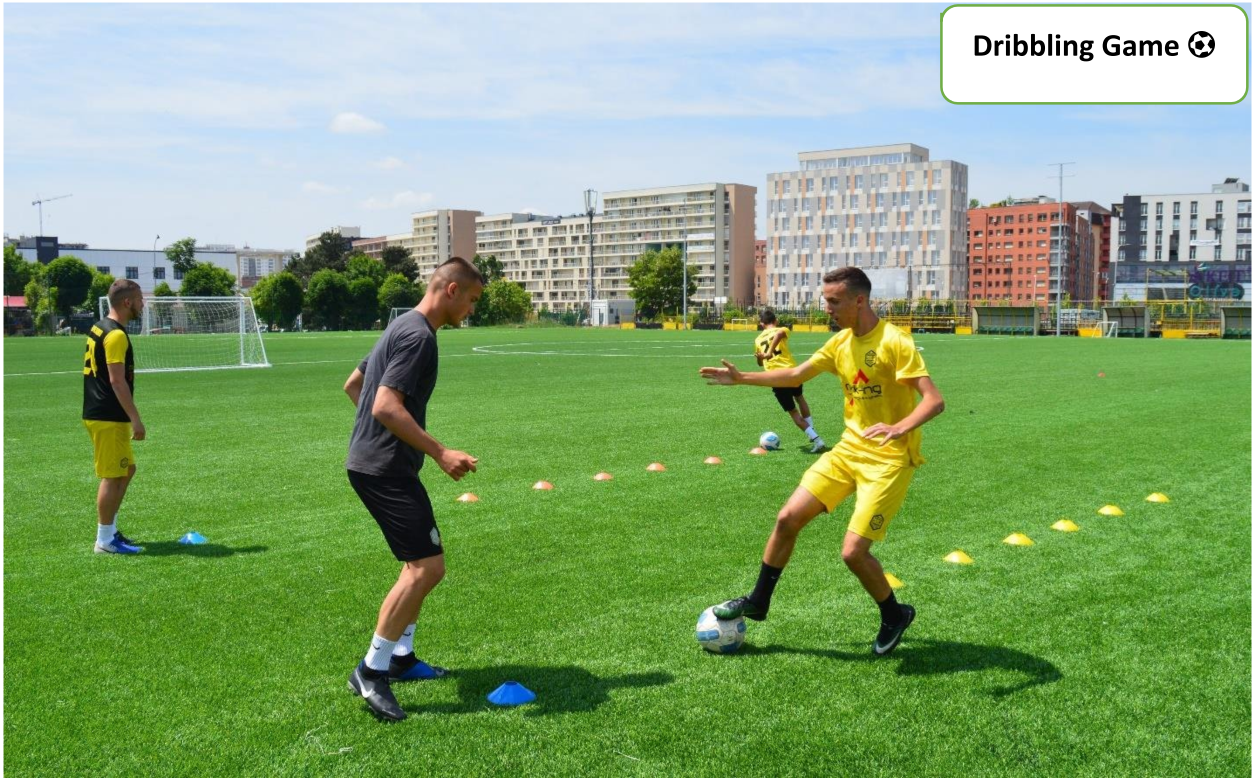

Dribbling Game
