# Supplementary figures and images for: Impact of the ‘FUNBALL’ Programme on Severe Injuries Among Young Male Football Players: A Secondary Analysis from a Cluster-Randomised Controlled Trial
Source: Sports Med Open. 2025 Nov 27;11:151. doi: 10.1186/s40798-025-00945-3 (PMC12660612; doi:10.1186/s40798-025-00945-3)

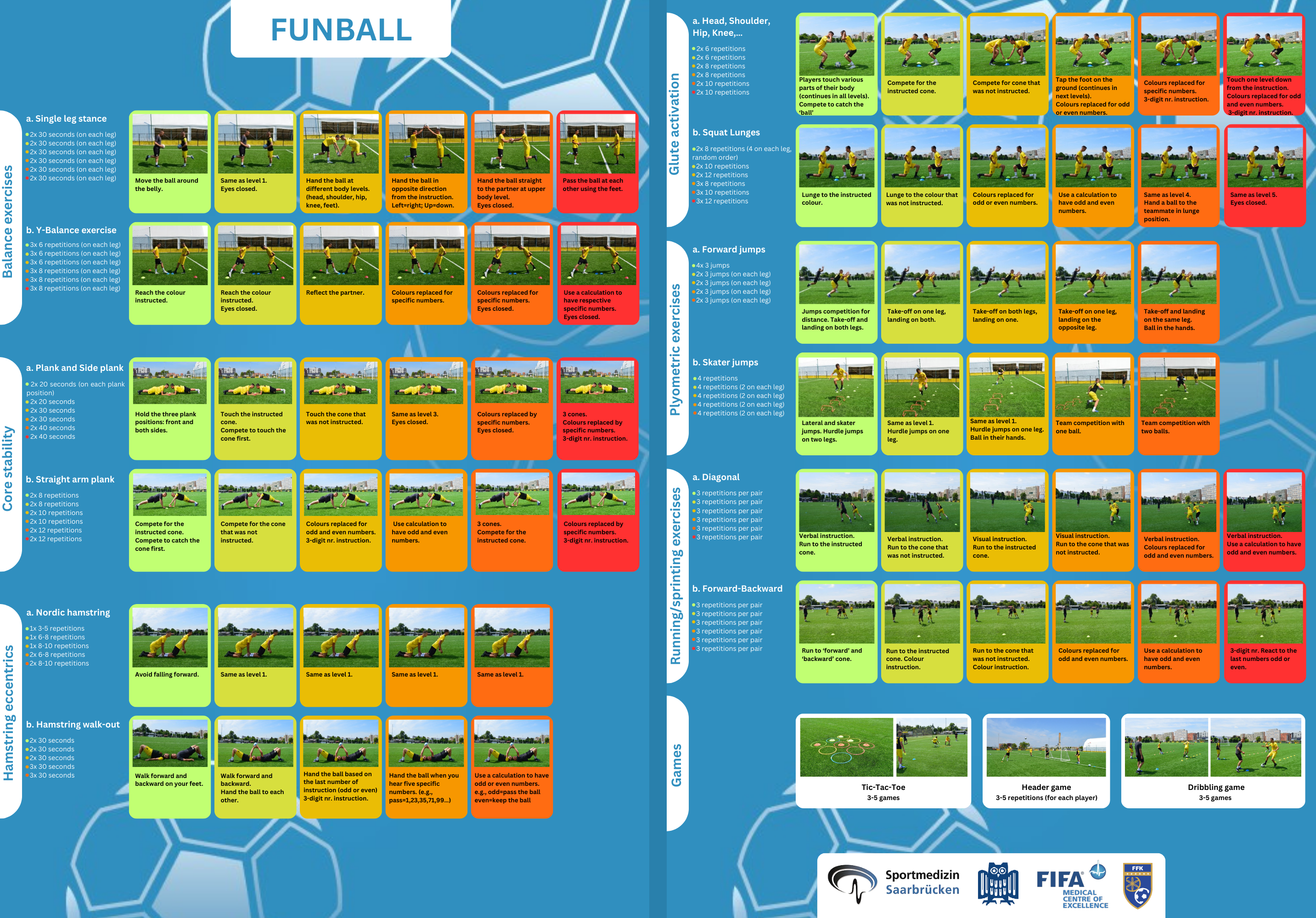

Supplement: Supplementary file 2 — Supplementary Material 2 [file 40798_2025_945_MOESM2_ESM.png]
