# Supplementary material for: Impact of the ‘FUNBALL’ Programme on Severe Injuries Among Young Male Football Players: A Secondary Analysis from a Cluster-Randomised Controlled Trial
Source: Sports Med Open. 2025 Nov 27;11:151. doi: 10.1186/s40798-025-00945-3 (PMC12660612; doi:10.1186/s40798-025-00945-3)
Supplement: Supplementary file 3 — Supplementary Material 3. [file 40798_2025_945_MOESM3_ESM.doc]

**Injury documentation**

1. ***a) Date of injury:****______________* ***b) Return to play:*** *______________*
2. ***a) Injuried body region:***

Head/Face  Shoulder/Clavicula  Hip/ Groin

Neck/Cervical  Upper arm  Thigh

Sternum/Ribs/ Upper back  Elbow  Knee

Abdomen  Forearm  Lower leg / Achilles tendon
 Lower back/ Sacrum/ Pelvis  Wrist  Ankle

Hand / Finger / Thumb  Foot/ Toe

***b) Body side:***

right  left  not applicable

1. ***Type of injury:***

Concussion  Muscle injury (fiber tear / strain / cramp)  *(with or without unconsciousness)*   Tendon injury (tear / tendinosis / bursitis)

Fracture  Hematoma / Bruise / Effusion

Other bone injuries  Abrasion

Disloction, (Sub-)luxation  Cracks / bruises

Distorsion/ Ligament injury  Nerve injury

Cartilage / meniscus lesion  Tooth damage

Other:_____________________________

1. Exact diagnosis and localization (text)__________________________________________­_____

_____________________________________________________________________________

1. a) When did the player get the injury?

Training  Match  Other (e.g. free time)

b) Injury ocurred ?

Beginning of training/match  End of training/match

1. What was the cause of the injury?

Overuse  Trauma

1. What caused the injury ?

**Contact** with another player  **Without** contact with another player

Foul against injured player  Change of direction

Fouling another player  Running / Jumping / Shooting

Duel  The ball

Other (please specify):_______________________________________________

1. *8. Has the player previously had such an injury on the same part of the body ?*

No  Yes, _______months ago

*If YES, when was the player fully operational again?* _______________________________
